# Plastome evolution in Santalales involves relaxed selection prior to loss of *ndh* genes and major boundary shifts of the inverted repeat

**DOI:** 10.1093/aob/mcae145

**Published:** 2024-08-30

**Authors:** Maja Edlund, Benjamin M Anderson, Huei-Jiun Su, Tanner Robison, Marcos A Caraballo-Ortiz, Joshua P Der, Daniel L Nickrent, Gitte Petersen

**Affiliations:** Department of Ecology, Environment and Plant Sciences, Stockholm University, SE-106 91 Stockholm, Sweden; Western Australia Herbarium, Department of Biodiversity, Conservation and Attractions, Locked Bag 104, Bentley Delivery Centre, 6983, Australia; Department of Earth and Life Sciences, University of Taipei, Taipei 100234, Taiwan; 121 Boyce Thompson Institute, Ithaca, NY 14853, USA; Plant Biology Section, School of Integrative Plant Science, CALS, Cornell University, Ithaca, NY 14853, USA; Department of Botany, National Museum of Natural History, Smithsonian Institution, MRC-166, PO Box 37012, Washington, DC 22013-7012, USA; Department of Biological Science (MH-282), California State University, Fullerton, PO Box 6850, Fullerton, CA 92834-6850, USA; Plant Biology Section, School of Integrative Plant Science, CALS, Cornell University, Ithaca, NY 14853, USA; Department of Ecology, Environment and Plant Sciences, Stockholm University, SE-106 91 Stockholm, Sweden

**Keywords:** Gene loss, inverted repeat, parasitism, phylogeny, plastome evolution, Santalales

## Abstract

**Background and Aims:**

Biological aspects of haustorial parasitism have significant effects on the configuration of the plastid genome. Approximately half the diversity of haustorial parasites belongs to the order Santalales, where a clearer picture of plastome evolution in relation to parasitism is starting to emerge. However, in previous studies of plastome evolution there is still a notable under-representation of members from non-parasitic and deep-branching hemiparasitic lineages, limiting evolutionary inference around the time of transition to a parasitic lifestyle. To expand taxon sampling relevant to this transition we therefore targeted three families of non-parasites (Erythropalaceae, Strombosiaceae and Coulaceae), two families of root-feeding hemiparasites (Ximeniaceae and Olacaceae) and two families of uncertain parasitic status (Aptandraceae and Octoknemaceae). With data from these lineages we aimed to explore plastome evolution in relation to the evolution of parasitism.

**Methods:**

From 29 new samples we sequenced and annotated plastomes and the nuclear ribosomal cistron. We examined phylogenetic patterns, plastome evolution, and patterns of relaxed or intensified selection in plastid genes. Available transcriptome data were analysed to investigate potential transfer of *infA* to the nuclear genome.

**Results:**

Phylogenetic relationships indicate a single functional loss of all plastid *ndh* genes (*ndhA*–*K*) in a clade formed by confirmed parasites and Aptandraceae, and the loss coincides with major size and boundary shifts of the inverted repeat (IR) region. Depending on an autotrophic or heterotrophic lifestyle in Aptandraceae, plastome changes are either correlated with or pre-date the evolution of parasitism. Phylogenetic patterns also indicate repeated loss of *infA* from the plastome, and based on the presence of transcribed sequences with presequences corresponding to thylakoid luminal transit peptides, we infer that the genes were transferred to the nuclear genome.

**Conclusions:**

Except for the loss of the *ndh* complex, relatively few genes have been lost from the plastome in deep-branching root parasites in Santalales. Prior to loss of the *ndh* genes, they show signs of relaxed selection indicative of their dispensability. To firmly establish a potential correlation between *ndh* gene loss, plastome instability and evolution of parasitism, it is pertinent to refute or confirm a parasitic lifestyle in all Santalales clades.

## INTRODUCTION

Parasitism in land plants can be divided into two main categories: mycoheterotrophy, where the plant relies on a fungal connection to its host to mediate uptake of nutrients, and haustorial parasitism, where nutrient uptake is mediated directly through a specialized haustorium (a parasitic root) formed by the plant itself (Leake, 1994; Nickrent, 2020). With increasing levels of heterotrophism and increased carbon uptake from the host, reliance on independent carbon acquisition is reduced. In holoparasites, photosynthesis has been lost completely, whereas hemiparasites have retained a capacity to perform photosynthesis (Westwood *et al*., 2010).

Haustorial parasitism has evolved independently at least 12 times within the flowering plants, but of these only two lineages, i.e. the family Orobanchaceae and the order Santalales, display the full span of life forms from non-parasites through hemiparasites to holoparasites (Nickrent, 2020). The sandalwood order (Santalales) is the largest group, including more than half of all described haustorial parasitic species, and it is the only group that also includes both root- and stem-feeding parasites (Nickrent, 2020). Thus, Santalales offers an excellent framework for studies of molecular evolution both during the transition from a non-parasitic to a parasitic lifestyle and during the transition between types of parasitism.

The relaxed dependence on photosynthesis is correlated with a degradation of the plastome, including structural changes, elevated substitution rates, pseudogenization, gene loss, and general size reductions of both coding and non-coding regions (Wicke *et al.*, 2016; Graham *et al.*, 2017; Wicke and Naumann, 2018). Degradation of the plastome seems to typically follow a convergent trajectory in heterotrophic or partially heterotrophic lineages, correlated with the degree of host dependence, where hemiparasites and partial mycoheterotrophs tend to have less-degraded plastomes compared to holoparasites and full mycoheterotrophs (Wicke *et al.*, 2016; Graham *et al.*, 2017; Wicke and Naumann, 2018).

In the typically less-degraded plastomes of hemiparasites and partial mycoheterotrophs, functional loss of the plastid-encoded *ndh* genes seems to be one of the most apparent and unifying traits (Wicke *et al.*, 2011; Graham *et al.*, 2017; Wicke and Naumann, 2018; Chen *et al.*, 2020). However, an exception is the hemiparasitic Krameriaceae with intact *ndh* genes (Banerjee *et al*., 2022). The plastid *ndh* genes code for some of the subunits of the NADH dehydrogenase-like (NDH) complex, whereas additional subunits are nuclear encoded (Shikanai, 2020; Ma *et al.*, 2021). It was proposed that *ndh* genes lost from the plastome could be transferred to the nuclear (or mitochondrial) genome (Lin *et al*., 2015), but instead a correlated loss of plastid and nuclear genes suggesting complete loss of the NDH complex has been described (Lin *et al.*, 2017). Curiously, recurrent loss of plastid *ndh* genes is observed not just in parasitic and other heterotrophic plants, but even in a number of autotrophs (see Graham *et al.*, 2017; Wicke and Naumann, 2018; Strand *et al.*, 2019). While the loss of *ndh* genes in holoparasites and full mycoheterotrophs is expected due to complete loss of photosynthesis, a similar loss in hemiparasites, partial mycoheterotrophs and carnivorous plants has been associated with the source or assimilation of nutrients (e.g. Wicke *et al.*, 2011; Nevill *et al.*, 2019). For autotrophic plants, a number of hypotheses have been proposed to explain *ndh* gene loss, but the underlying factors remain obscure (Strand *et al*., 2019). Thus, it is relevant to ask how, if at all, the loss is related to the evolution of a heterotrophic lifestyle.

Within Santalales, a continuously more comprehensive picture of plastome configuration and evolution is starting to form, although many published papers include data from just a single or a few plastomes (Petersen *et al.*, 2015; Zhang *et al.*, 2015; Su and Hu, 2016; G.-S. Yang *et al.*, 2017; Li *et al.*, 2017; Shin and Lee, 2018; Zhu *et al.*, 2018; Guo and Ruan, 2019*a*, *b*; Guo *et al.*, 2019, 2020, 2021*a*, *b*; Jiang *et al.*, 2019; S.-S. Liu *et al.*, 2019; Schelkunov *et al.*, 2019; Su *et al.*, 2019, 2021; Y. Yang and He, 2019; Yu *et al.*, 2019; Chen *et al.*, 2020; D. Yang *et al*., 2020; Ceriotti *et al.*, 2021; Nickrent *et al.*, 2021; X. Liu *et al*., 2021; Al-Juhani *et al.*, 2022; Darshetkar *et al*., 2023; Tang *et al.*, 2024). In the most comprehensive study to date (Chen *et al.*, 2020), most genomic changes were found to be associated with the evolution of hemi- and holoparasitism, and only minor differences were found between root- and stem-feeding hemiparasites. Loss of *ndh* genes was found in all parasites, but not in non-parasites. However, in their study taxon sampling of non-parasitic and root-feeding hemiparasitic lineages was limited and may have resulted in a simplified picture of plastome evolution. Thus, deeper sampling from those clades is strongly called for.

**Fig. 1.**
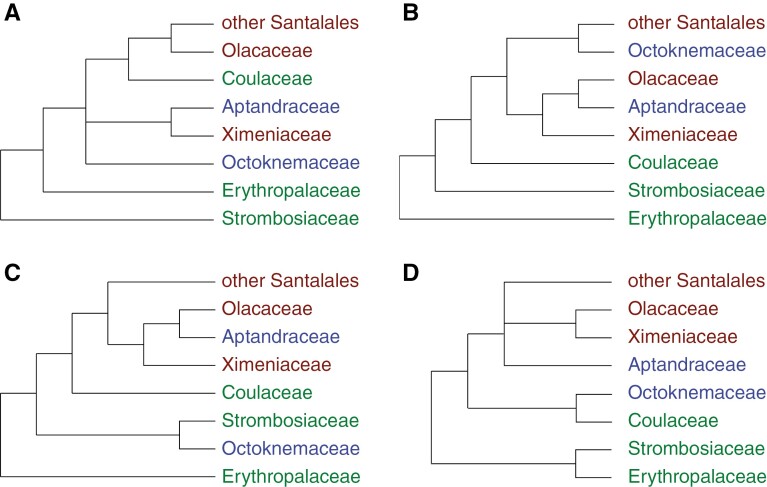
Four alternative topologies from previous and present phylogenetic analyses of Santalales: (A) Su *et al.* (2015), 3 plastid + 1 mitochondrial + 3 nuclear genes; (B) Nickrent *et al.* (2019), 3 plastid + 2 nuclear genes; (C) Nickrent (2020), 3 plastid + 1 mitochondrial + 3 nuclear genes; (D) this study, 76 plastid + 3 nuclear genes, summary tree of maximum likelihood and Bayesian inference analyses. Green terminals are non-parasitic; brown terminals are parasitic; blue terminals have uncertain parasitic status. Other Santalales comprise 13 families including root and stem hemiparasites as well as holoparasites.

To trace plastome evolution in Santalales, a solid phylogenetic framework is needed. Despite ambiguity in the sister group relationship of the order (APG IV, 2016; Leebens-Mack *et al.*, 2019; Baker *et al.*, 2022, Zuntini *et al.*, 2024), phylogenetic relationships within Santalales are becoming increasingly clear, and the monophyly of clades at the level of family (*sensu*  Nickrent *et al.*, 2010) is mostly well supported (Su *et al.*, 2015; Nickrent *et al.*, 2019). However, some ambiguity remains about the deepest splits involving non-parasitic and root-feeding, hemiparasitic families (Fig. 1).

Tracing the early evolutionary steps towards parasitism is further complicated by missing data of the potential parasitic status of several taxa (Kuijt and Hansen, 2015). Whereas the parasitic status of holoparasites and stem-feeding parasites is clear, a potential root-feeding parasitic habit remains to be firmly established for some taxa. As delineated here, Strombosiaceae, Erythropalaceae and Coulaceae are generally considered non-parasitic, while the status of Aptandraceae and Octoknemaceae is insufficiently known (Kuijt and Hansen, 2015; Nickrent, 2020) (Fig. 1). Though not all genera within Ximeniaceae and Olacaceae have been investigated, both families are generally considered parasitic based on the presence of secondary haustoria in the roots of *Ximenia* (Ximeniaceae; DeFilipps, 1969), *Olax* (Olacaceae; Barber, 1907) and *Ptychopetalum* (Olacaceae; Anselmino, 1932).

Attempting to trace the early steps of plastome evolution in Santalales, we here target the families of assumed non-parasitic status (Strombosiaceae, Erythropalaceae and Coulaceae) and of unclear parasitic status (Aptandraceae and Octoknemaceae). Further, we add data to two families of root-feeding hemiparasites, Ximeniaceae and Olacaceae, the latter not previously being included in studies of complete plastome evolution. Based on 29 newly sequenced plastomes and the nuclear ribosomal DNA cistron region from Santalales, we complement previous studies of phylogenetic patterns and plastome evolution with an emphasis on the transition to a parasitic lifestyle. In general, we explore whether plastome configuration and gene content is affected by the transition to parasitism, and in particular whether the loss of *ndh* genes can be correlated with this shift or possibly occurred prior to it. We also investigate whether changes in selection of genes or gene complexes can be related to shifts in lifestyle.

## MATERIALS AND METHODS

### Taxon sampling

Taxon sampling was guided by previous studies of the order (Malécot and Nickrent, 2008; Su *et al.*, 2015; Nickrent *et al.*, 2019), with a focus on non-parasitic and root hemiparasitic lineages. Following family delineation in Nickrent *et al.* (2010, 2019), we sampled three species from Erythropalaceae, six from Strombosiaceae, six from Aptandraceae, one from Octoknemaceae, two from Ximeniaceae, three from Olacaceae and seven from the remaining families of the order, thereby allowing a comparative framework that includes all major photosynthetic lineages in the order (see Supplementary Data Table S1). Three previously published Santalalean plastomes were included in the study: *Erythropalum scandens* (NC036759, Erythropalaceae), *Malania oleifera* (MK764537, Ximeniaceae) and *Loranthus europaeus* (MT987630, Loranthaceae). Four plastomes from taxa outside Santalales were downloaded from GenBank to be used as outgroups: *Crataegus marshallii* (MK920293, Rosales: Rosaceae), *Helianthus annuus* (NC007977, Asterales, Asteraceae), *Myoporum bontioides* (NC050956, Lamiales, Scrophulariaceae) and *Silene kiusiana* (NC048886, Caryophyllales: Caryophyllaceae).

For the nuclear ribosomal cistron (hereafter nrDNA), we downloaded outgroup sequences from GenBank for *Crataegus marshallii* (MN215977), *Helianthus annuus* (KF767534), *Myoporum tetrandrum* (MN411551) and *Silene nutans* (MT735331). Outgroups were chosen to broadly cover a large section of angiosperm lineages, since the sister group relationship of Santalales is not clear (APG IV, 2016; Leebens-Mack *et al.*, 2019; Baker *et al.*, 2022; Zuntini *et al.*, 2024), and the selection was further guided by data availability of both plastome and nrDNA sequences in GenBank. For ingroup taxa, nrDNA sequences were downloaded for *Diogoa zenkeri* (MH390535, Strombosiaceae) and *Strombosia pustulata* (MH390536, Strombosiaceae) to extend incomplete sequences obtained in the present study. The two GenBank sequences were derived from the same specimens sequenced in this study.

### DNA extraction and sequencing

Genomic DNA was extracted from leaf material from either herbarium specimens or silica-dried fresh material, using a modified CTAB protocol (Doyle and Doyle, 1987). Library preparation was carried out with TruSeq DNA PCR-free libraries and sequenced as 150- or 250-bp paired-end reads on an Illumina NovaSeq 6000 at SciLifeLab Stockholm Campus Solna or NGS Core at National Taiwan University.

### Assembly of plastomes and the nuclear ribosomal cistron (nrDNA)

Assemblies and annotations were performed at three different laboratories following slightly different strategies. For the majority of samples, raw paired-end reads were trimmed for quality (minimum Phred score of 20, minimum length of 50 bp) and adapter contamination with BBDuk, duplicates were removed with Clumpify, and overlapping reads were merged with Tadpole (after extension and recalculation of Phred scores) from BBMap v.38.81 (Bushnell, 2014). This resulted in two sets of reads (merged and unmerged) for downstream processing.

To avoid contamination from other genomic compartments and to get a manageable dataset, trimmed reads were mapped against an appropriate reference (plastome of the putative closest available relative) from GenBank (Supplementary Data Table S1). Reads matching the corresponding k-mer depth were then extracted using KAT v.2.4.2 (Mapleson *et al.*, 2017). This subset of reads was used for *de novo* assembly in Unicycler v.0.4.9b (Wick *et al.*, 2017). Due to low read numbers in some cases, kmer-filtering was not performed on all read-sets (see Table S1). Bandage v.0.8.1 (Wick *et al.*, 2015) was used to view and evaluate assemblies, and to assess contig connections. When the expected structure of a small and a large contig connected at both ends to a higher-depth linear contig [representing the small and large single-copy regions, connected to the inverted repeat (IR) region, respectively] was found, the plastome was circularized. If not, further extension was carried out with Tadpole (with the settings *k* = 62, extend left and right = 10 000) on the subset of contigs matching plastome blast hits from a local database (as implemented in Bandage). The extended contigs were used as a reference for mapping reads for another iteration of Unicycler *de novo* assembly. This procedure was repeated until either the plastome was circularized or no further extension could be achieved. The resulting plastome assemblies were quality checked by mapping the KAT-filtered plastome reads (or the full set of quality trimmed reads for the smaller read-sets) in Geneious Prime v.2019.0.4 (Biomatters, Auckland, New Zealand) with default settings for manual inspection, and a minimum read-depth of 7 (without conflict) in any position was considered reliable.

Most plastome annotations were carried out in GeSeq (Tillich *et al.*, 2017) through the online application (https://chlorobox.mpimp-golm.mpg.de/geseq.html). Settings were left unchanged except for ‘protein search identity’ which was changed to 75, and the third-party programs tRNAscan-SE v.2.0.7. (Chan and Lowe, 2019) and Chloë v.0.1.0. (https://chloe.plantenergy.edu.au) were also used. Annotation duplications were removed, generally favouring Chloë annotations, as they more often resulted in annotations of the expected length and position of the genes (in comparison to previously published plastomes).

For *Misodendrum brachystachyum* (Misodendraceae), raw reads were trimmed as above and the plastome was *de novo* assembled and circularized using NOVOPlasty v.2.7.2 (Dierckxsens *et al.*, 2017) using the *rbcL* sequence from *Malania oleifera* as seed and a k-mer of 33. Annotations were performed using the Transfer annotations tool in Geneious Prime, where genes were predicted based on amino acid sequence similarity to *Malania oleifera* (MK764537). Assembly and annotation of *Olax imbricata* and *Scorodocarpus borneensis* (Strombosiaceae) plastomes were performed using strategies previously described (Su *et al.*, 2021).

To identify potential structural differences, whole-plastome alignments were performed using Mauve v.1.1.3 (http://darlinglab.org/mauve/mauve.html) as implemented in Geneious Prime 2024.0.3 (https://www.geneious.com).

The nrDNA region was assembled following a procedure similar to the main one described above, with the exception that kmer depth was evaluated based on 18S, 26S, and (when available) internal transcribed spacer (ITS) and/or 5.8S contigs downloaded from GenBank (see Supplementary Data Table S1).

### Alignments and phylogenetic analysis

One copy of each (translated) coding sequence (CDS) region from the plastome was extracted and aligned separately using MAFFT v.7.310 (Katoh and Standley, 2013) with the L-INS-I setting applied. After alignment, CDS regions were converted back to their corresponding nucleotide sequences with pal2nal v.14 (Suyama *et al.*, 2006), producing aligned DNA sequences. Sites with more than 50 % gaps were removed from all alignments. After visual inspection in alan alignment viewer v.2.1.0 (https://www.github.com/mpdunne/Alan), two gene alignments (*rpl22*, *rps16*) were excluded due to poor alignment before concatenation and partitioning (with separate partitions kept for regions and codon positions). For the nrDNA, 18S, 5.8S and 26S nucleotide sequences were aligned with MAFFT, trimmed as above and partitioned by region, only. The ITS regions were not used because of considerable alignment ambiguity. Scripts for trimming, concatenation and partitioning are available on a Github repository (https://github.com/bmichanderson/scripts).

A maximum likelihood analysis with automatic model selection for all partitions (Kalyaanamoorthy *et al.*, 2017) and partition merging (Lanfear *et al*., 2012) was implemented in IQ-TREE v.2.1.3. (Minh *et al.*, 2020), executing ten runs (‘--runs 10’) with 10 000 ultrafast bootstrap replicates (Hoang *et al.*, 2018), which re-sampled partitions and sites within them [with the flag ‘--sampling GENESITE’ (Gadagkar *et al.*, 2005; Seo *et al.*, 2005)]. Analyses were carried out separately for the plastome and nrDNA. As no strongly supported conflict (ultrafast bootstrap, UB > 95) was observed between the resulting trees (Supplementary Data Figs S1 and S2), a combined analysis of both plastome and nrDNA data sets was carried out with the same settings as above to improve phylogenetic signal.

To complement the maximum likelihood analyses, the same alignments were analysed using a Bayesian approach in MrBayes v.3.2.7 (Ronquist *et al.*, 2012). To determine the optimum partitioning and models for the plastid, nuclear and combined alignments, these were run again with IQ-TREE using the options ‘-m TEST -mset mrbayes’ to restrict the search to models that could be implemented in MrBayes. The resulting optimal models were then implemented with priors fixed to the values determined in the IQ-TREE model search. The Bayesian analyses were run until the average standard deviation of split frequencies between the two independent runs per analysis was <0.005 or the run reached 500 000 generations (sampling every 1000 generations), whichever took longer. Estimated sample size (ESS) values for tree length were >300 for all analyses, and the potential scale reduction factors (PSRFs) were all close to 1, indicating a good sampling of the posterior and convergence of the independent runs. A relative burn-in of 25 % was used for diagnostics and sampling parameters and trees. The majority-rule consensus tree for each analysis was displayed using FigTree v.1.4.4 (Rambaut, 2018; https://github.com/rambaut/figtree). To visualize differences between trees in the credible sets of trees, consensus networks (25 % cutoff for displaying splits) were constructed using SplitsTree4 v.4.17.1 (Huson and Bryant, 2006).

Functional gene losses were mapped on the phylogenetic tree derived from the combined maximum likelihood analysis. Mapping was based on the principle of parsimony and the assumption that genes may be lost but not gained, and that pseudogenes do not revert to functional copies.

### Selection strength changes

Signs of relaxed or intensified selection pressures in protein coding genes were investigated utilizing RELAX (Wertheim *et al.*, 2015), as implemented in HyPhy v.2.5.48 (Pond *et al.*, 2005). RELAX investigates if the ratio of non-synonymous to synonymous substitutions (dN/dS or ω, where ω > 1 indicates positive selection and ω < 1 indicates purifying selection) in a specified set of test branch/es of the tree differs from a set of reference branch/es. A selection intensity parameter, *k*, is calculated, where *k* > 1 indicates relatively intensified selection and *k* < 1 indicates relatively relaxed selection (Wertheim *et al.*, 2015). This was performed on functional gene groups, as well as for separate *ndh* genes and other individual genes, which cannot be assigned to a functional group. Terminal taxa were treated as individual test branches, against the four non-Santalalean outgroup taxa assigned as the reference group (remaining taxa left undefined). Alignments from the phylogenetic inference were used, ensuring correct codon positioning and interpretation, with alignments concatenated by each functional gene group (e.g. *atpA*–*I* concatenated for the *atp* group). Trees were inferred for each group or gene separately using the same settings as for the main phylogenetic analyses, except only 1000 ultrafast bootstrap replicates were used and sampled by site only. Branches with <50 % ultrafast bootstrap support were collapsed to polytomies with Newick Utilities (Junier and Zbodnov, 2010). RELAX was run separately for each test terminal (up to 32 per alignment/tree) for 30 gene group alignments/trees (>700 runs) in parallel as a batch job on the Pawsey Setonix supercomputer (Perth, Western Australia). The entire set of runs was repeated five times after detecting issues with convergence, i.e. in a few cases significantly different values of *k* were obtained. To improve convergence, RELAX was run with the options ‘--starting points 10 --models Minimal’ following advice from the developer (Sergei Pond, pers. comm.). Increasing the number of starting points from one (the default) makes RELAX perform more initial optimizations to estimate a good starting point for complete model optimization. The ‘--models Minimal’ flag makes RELAX skip calculating the parameter-rich General Descriptive model, which in some cases may improve convergence (S. Pond, pers. comm.). For each test, the *k* value with the highest likelihood across the five runs was reported along with the significance compared to the alternative model.

### Transfer of infA to the nuclear genome

To detect a possible transfer of *infA* from the plastome to the nuclear genome within Santalales, the *infA* gene from *Nuytsia floribunda* (MT987640, Loranthaceae) was blasted against a local database of ~210 000 assembled transcripts from *Viscum album* (Viscaceae; Petersen *et al.*, 2022) using the BLASTN algorithm as implemented in Geneious Prime v.2020.2.2 (Biomatters Ltd). The same *infA* sequence from *Nuytsia* was also blasted against the whole-genome shotgun contig library of *Santalum album* (Bioproject PRJNA422746, Santalaceae) and the transcriptome shotgun assembly library of *Taxillus chinensis* (Bioproject PRJNA548287, Loranthaceae) available in GenBank. These species were selected because all lack the *infA* gene in their plastomes (Petersen *et al.*, 2015; Guo *et al.*, 2020; Su *et al.*, 2021). Since the identified nuclear *infA* sequences (from start to stop codon) were more than twice as long as *infA* plastome genes from Santalales, we tested if the longer N-terminal region could be a presequence coding for a transit peptide using TargetP-2.0 (Almagro Armenteros *et al.*, 2019). The (translated) nuclear genes (*infA*-N) and plastome genes (*infA*-P) of *Nuytsia floribunda* (accession MT987640) and the newly generated *Schoepfia arenaria* (Schoepfiaceae) were aligned with MAFFT v.7.310 (with the L-INS-i setting applied) for comparison.

## RESULTS

### Plastome characteristics

**Fig. 2.**
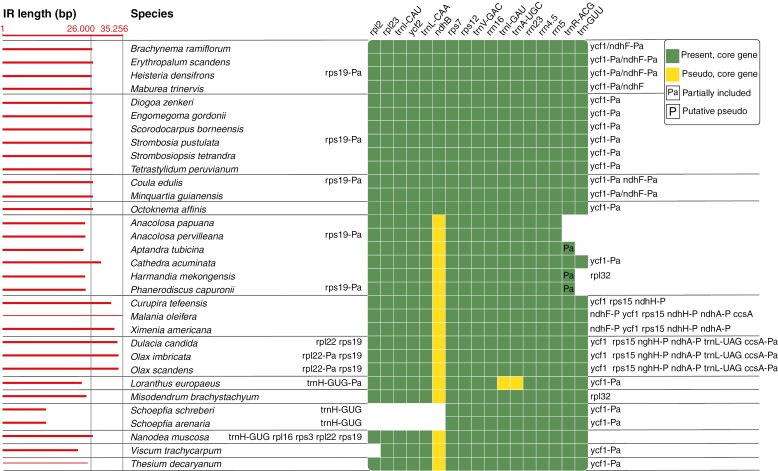
Inverted repeat (IR) region in representatives of Santalales. Red bars showing relative length of the IR. Coloured squares indicate a core IR region shared by outgroup representatives and ancestral Santalales. Functional genes in the core IR region are in green, pseudogenes in yellow. Genes of species with expanded IRs are listed upstream or downstream of the core region. Pa = genes partially included in the IR; P = pseudogenes in the non-core IR region.

New plastome sequence data were generated for 29 species (Supplementary Data Table S2). The number of trimmed reads varied from 3 697 838 to 146 256 820, resulting in an average read-depth of plastomes of 32–6909.2 and an average read-depth of nrDNA regions of 430.9–14 264.2. For 21 samples, complete, circular plastomes were assembled. The remaining eight samples, where read-depth was typically lower, were assembled into 3–14 contigs (Table S2). The total length of all completely assembled Santalales plastomes varied from 122 120 bp (*Schoepfia schreberi*) to 161 120 bp (*Minquartia guianensis*, Coulaceae). They all had the quadripartite structure typical of angiosperm plastomes, with a GC content of ~35–38 % (Table 1). The length of the IR varied from 12 426 bp (*Schoepfia schreberi*) to 35 256 bp (*Malania oleifera*) (Table 1). The IR size differences were typically connected to movement of the borders between the IRs and the single-copy regions, causing inclusion or exclusion of genes from the IR (Fig. 2).

**Table 1.** Characteristics of plastomes of Santalales. Since complete, circular assemblies were not obtained for all samples, the number of assembled contigs is given. A complete circular assembly has one contig.

Family	Species	Plastome size (bp)	GC (%)	IR size (bp)	Genes			Contigs
					Protein	rRNA	tRNA	
Erythropalaceae	*Brachynema ramiflorum*	>129 255	~36.7	26 160	78	4	28	14
Erythropalaceae	*Erythropalum scandens*	156 154	38	26 394	79	4	30	1
Erythropalaceae	*Heisteria densifrons*	>159 896	~37.4	26 046	79	4	30	7
Erythropalaceae	*Maburea trinervis*	>128 892	~36.6	26 069	79^1^	4	30	5
Strombosiaceae	*Diogoa zenkeri*	159 584	37.7	26 250	79	4	30	1
Strombosiaceae	*Engomegoma gordonii*	159 618	37.7	26 245	79	4	30	1
Strombosiaceae	*Scorodocarpus bornensis*	159 272	37.8	26 136	79	4	30	1
Strombosiaceae	*Strombosia pustulata*	159 388	37.8	26 215	79	4	30	1
Strombosiaceae	*Strombosiopsis tetrandra*	160 070	37.6	26 119	79	4	30	1
Strombosiaceae	*Tetrastylidium peruvianum*	>158 467	~37.9	26 191	79	4	30	5
Coulaceae	*Coula edulis*	>121 474	~36.8	26 382	79	4	29	3
Coulaceae	*Minquartia guianensis*	161 120	37.7	26 340	79	4	30	1
Octoknemaceae	*Octoknema affinis*	160 640	37.5	26 325	79	4	30	1
Aptandraceae	*Anacolosa papuana*	149 924	36.9	24 090	68	4	30	1
Aptandraceae	*Anacolosa pervilleana*	150 027	37.1	24 167	68	4	30	1
Aptandraceae	*Aptandra tubicina*	143 301	36.9	23 592	68	4	28	1
Aptandraceae	*Cathedra acuminata*	156 432	36.8	28 758	68	4	30	1
Aptandraceae	*Harmandia mekongensis*	141 146	37.4	24 055	68	4	29	1
Aptandraceae	*Phanerodiscus capuronii*	>127 374	~35.5	24 234	67	4	30	12
Ximeniaceae	*Curupira tefeensis*	>122 618	~35.8	31 713	68	4	30	5
Ximeniaceae	*Malania oleifera*	158 163	36.7	35 256	70	4	30	1
Ximeniaceae	*Ximenia americana*	156 507	36.8	32 746	69	4	30	1
Olacaceae	*Dulacia candida*	156 333	35.6	33 606	67	4	30	1
Olacaceae	*Olax imbricata*	155 798	35.8	33 982	67	4	30	1
Olacaceae	*Olax scandens*	>156 106	~35.8	33 933	67	4	29	3
Schoepfiaceae	*Schoepfia arenaria*	122 210	37.9	12 438	69	4	30	1
Schoepfiaceae	*Schoepfia schreberi*	122 120	37.9	12 426	71	4	29	1
Misodendraceae	*Misodendrum brachystachyum*	135 241	37.1	24 485	70	4	28	1
Loranthaceae	*Loranthus europaeus*	123 004	37.1	23 087	66	4	28	1
Thesiaceae	*Thesium decaryanum*	141 066	36.5	25 031	67	4	30	1
Nanodeaceae	*Nanodea muscosa*	140 372	37.5	26 317	68	4	30	1
Viscaceae	*Viscum trachycarpum*	128 593	36.8	21 907	67	4	29	1

^1^ *ndhF* partial.

Gene content of all complete plastomes included in the study varied from 98 (*Loranthus europaeus*, Loranthaceae) to 113 (most non-parasitic species), with 66–79 protein coding genes, four rRNA genes and 28–30 tRNA genes (Table 1). The gene order was conserved within the order, and no genome rearrangements were observed among the complete plastomes. For the eight species where complete plastomes could not be assembled, rearrangements may exist and a few genes may not have been detected (Supplementary Data Table S3).

The difference in gene numbers observed among the sampled Santalales is mostly due to absence or presence of functional copies of the *ndh* genes. In Erythropalaceae, Strombosiaceae, Coulaceae and Octoknemaceae, all 11 genes (*ndhA*–*K*) are present and presumably functional, but in the remaining taxa only pseudogenes or short gene fragments could be identified (Table 2).

**Table 2.** Presence of *ndh* pseudogenes or gene fragments (•) in species of Santalales.

Family	Species	*ndhA*	*ndhB*	*ndhC*	*ndhD*	*ndhE*	*ndhF*	*ndhG*	*ndhH*	*ndhI*	*ndhJ*	*ndhK*
Aptandraceae	*Anacolosa papuana*	•	•	•	•				•	•	•	•
	*Anacolosa pervilleana*	•	•	•	•				•	•	•	•
	*Aptandra tubicina*	•	•		•				•	•	•	
	*Cathedra acuminata*	•	•	•	•			•	•	•	•	•
	*Harmandia mekongensis*		•						•	•	•	
	*Phanerodiscus capuronii*	•	•		•			•	•	•		
Ximeniaceae	*Curupira tefeensis*		•		•				•			
	*Malania oleifera*	•	•				•		•	•		
	*Ximenia americana*	•	•		•		•		•	•		
Olacaceae	*Dulacia candida*	•	•						•			
	*Olax imbricata*	•	•		•				•			
	*Olax scandens*	•	•		•		•		•			
Schoepfiaceae	*Schoepfia arenaria*	•		•	•			•	•	•	•	•
	*Schoepfia schreberi*	•		•	•			•	•	•	•	•
Misodendraceae	*Misodendrum brachystachyum*		•		•						•	
Loranthaceae	*Loranthus europaeus*		•									
Thesiaceae	*Thesium decaryanum*	•	•									
Nanodeaceae	*Nanodea muscosa*	•	•								•	
Viscaceae	*Viscum trachycarpum*		•									

### Phylogenetic results

**Fig. 3.**
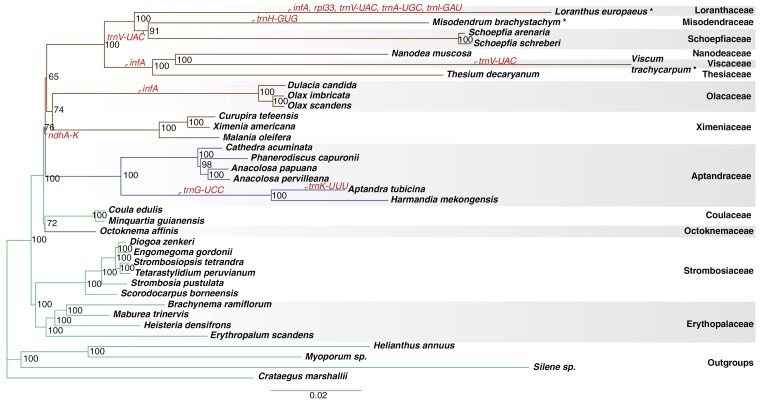
Phylogeny of Santalales based on maximum likelihood analysis of combined plastome gene and nrDNA sequences. Node values are ultrafast bootstrap support. Green branches correspond to non-parasitic lineages, brown branches to parasitic lineages. Blue branches are lineages with uncertain parasitic status. Asterisks (*) after species names indicate stem parasites. Functional gene losses are marked in red. The scale bar indicates inferred substitutions per site.

The concatenated plastome plus nrDNA matrix contained 72 907 sites of which 48 453 were variable. The maximum likelihood analysis resulted in a phylogenetic tree with strong support for monophyly of all families represented by more than one species (UB values of 100) (Fig. 3). Erythropalaceae is strongly supported as sister to Strombosiaceae (UB 100), and this clade is sister to the rest of Santalales. Coulaceae and Octoknemaceae form a weakly supported clade (UB 72), in a sister group position to the confirmed parasites and Aptandraceae (UB 76), but with weakly supported internal relationships among Aptandraceae, Ximeniaceae, Olacaceae and the remaining core parasites. Comparing the topologies of trees derived from separate analyses of plastid and nuclear data revealed differences, but no strongly supported conflict (Supplementary Data Figs S1 and S2).

Bayesian inference of the combined matrix used 17 partitions with either the GTR + GAMMA or GTR + I + GAMMA models. Average ESS for tree length was 373 and the PSRF was 1.003. The credible set of trees contained two trees. A separate analysis of the plastid data used 16 partitions with either the GTR + GAMMA or GTR + I + GAMMA models. Average ESS for tree length was 376 and the PSRF was 0.999. The credible set of trees contained 11 trees. The nuclear analysis used two partitions with the SYM + I + GAMMA or GTR + I + GAMMA models. Average ESS for tree length was 582 and the PSRF was 1.000. The credible set of trees contained 504 trees. The majority rule consensus trees from combined and separate analyses are shown in Supplementary Data Fig. S3.

Comparing the plastid and nuclear trees from Bayesian inference showed some support (low support in the maximum likelihood trees) for conflicting placement of the clade containing *Coula edulis*, *Minquartia guianensis* (both Coulaceae) and *Octoknema affinis* (Octoknemaceae). The plastid analysis resolved those taxa as a clade (0.98 posterior probability, pp) in a larger clade (1.00 pp) containing the confirmed parasites and Aptandraceae. The nuclear analysis showed little support (0.64 pp) for those taxa as a clade, but resolved them in a larger clade (0.99 pp) containing the autotrophic Erythropalaceae and Strombosiaceae. The combined analysis resolved them as a clade (1.00 pp) sister to the confirmed parasites and Aptandraceae (1.00 pp) as in the plastid analysis. Another grouping that differed between the plastid and nuclear trees is a sister relationship between Ximeniaceae and Olacaceae, which was supported in the plastid analysis (1.00 pp), but not in the nuclear analysis, where Ximeniaceae was resolved sister (0.99 pp) to the core parasites and Olacaceae was resolved sister (0.99 pp) to Aptandraceae.

The topology of the trees from the combined analyses using maximum likelihood compared to a Bayesian approach differed only in the relative branching order of the confirmed parasites and Aptandraceae, with the maximum likelihood providing low support (UB 65) for Ximeniaceae + Olacaceae (UB 74) sister to the core parasites, but the Bayesian analysis with low support (0.93 pp) for Aptandraceae sister instead. The credible set of trees in the Bayesian analysis of the combined alignment contained only two trees, one with Aptandraceae sister and the other with Ximeniaceae + Olacaceae sister to the core parasites (Supplementary Data Fig. S4).

### Gene evolution

Phylogenetic relationships suggest a single functional loss of all *ndh* genes in the common ancestor to a clade including confirmed parasites plus Aptandraceae (Fig. 3), although only a partial sequence of *ndhF* could be obtained from the incomplete plastome assembly of *Maburea trinervis* (Erythropalaceae). However, read depth was low near the IR–Small Single Copy (SSC) borders where the gene is located, and a complete copy was probably missed. Additional functional gene loss includes the protein coding genes *infA* and *rpl33* and six tRNA genes (*trnA-UGC*, *trnG-UCC*, *trnH-GUG*, *trnI-GAU*, *trnK-UUU*, *trnV-UAC*). All losses are confined to species within the parasitic clade and Aptandraceae, with repeated losses of *infA* and *trnV-UAC* (Fig. 3).

Results from testing for changes in selection pressure are presented in Table 3 as the *k* values with the highest likelihood obtained across five runs of RELAX. Data from each individual run and a summary of the variation between runs are provided as Supplementary Data Table S4. Compared to running RELAX analyses with default parameters, convergence among runs was increased. Thus, results from individual runs were consistent for the majority of tests, but we observed significant differences in four cases: *cemA* – *Brachynema ramiflorum* (Erythropalaceae) and the *rps* gene group – *Coula edulis*, *Maburea trinervis* and *Viscum trachycarpum*. In the 19 genes or gene groups tested for 32 species, 20 % of the results showed significant levels of relaxed selection and 3 % significant levels of intensified selection (Table 3). In general, the results give a mixed picture with only few groups of taxa showing a consistent pattern of relaxation or intensification of individual genes or functional groups of genes. The *ycf1* gene stands out as being under relaxed selection in most species. Significant relaxed selection pressures were also evident in the *atp*, *ndh* and *rpl* complexes of all Erythropalaceae, and *Erythropalum scandens* showed relaxed selection in almost all photosynthesis-associated gene complexes. A similar trend of relaxed selection was apparent in the photosynthesis-associated genes in stem-feeding parasitic genera (*Misodendrum*, *Loranthus*, *Viscum*), where all complexes show signs of relaxed selection in all species, except *rbcL* in *Viscum trachycarpum*. It is remarkable that *Loranthus europaeus* showed significant relaxation in all the genes tested apart from those coding for protease (*clpP*) and the conserved open reading frames *ycf1*, *ycf2*, *ycf3* and *ycf4.* Though missing in at least three lineages within the order, *infA* only showed significant signs of relaxed selection pressure in *Anacolosa papuana*, *Harmandia mekongensis* (both Aptandraceae) and *Loranthus europaeus*.

**Table 3.** Patterns of relaxed and intensified selection in 11 individual genes and eight functional gene groups. Reference group: *Crataegus marshallii*, *Helianthus annuus*, *Myoporum bontioides* and *Silene kiusiana*. Blue boxes indicate significant relaxed selection, red boxes indicate significant intensified selection. Values are the selection intensity parameter *k*. Significance levels for *k*: *P < 0.05, **P < 0.01, ***P < 0.001.

Test species	*accD*	*atp*	*ccsA*	*cemA*	*clpP*	*infA*	*matK*	*ndh*	*pet*	*psa*	*psb*	*rbcL*	*rpl*	*rpo*	*rps*	*ycf1*	*ycf2*	*ycf3*	*ycf4*
*Brachynema ramiflorum*	0.6	0.6***	0.6	0.3*	0.0***	13.1	0.2*	0.3***	0.9	0.8	0.8	0.0	0.2***	0.9	0.6**	–	0.3	1.2	0.4
*Erythropalum scandens*	0.7	0.2***	0.5	0.5	0.0***	0.6	0.8	0.5***	0.0*	0.7**	0.8*	0.8	0.7*	0.8	0.9	0.2***	1.2	0.8	0.2*
*Heisteria densifrons*	1.0	0.7*	1.7	0.0**	5.5*	22.0	0.3*	0.1***	0.8	24.2	0.9	0.3	0.3**	0.3**	0.7	1.2	0.9	0.4	1.3
*Maburea trinervis*	1.9	0.6*	0.5	0.1	0.0	13.1	0.4*	0,5***	11.5*	1.0	0.6**	1.2	0.3*	0.4**	0.2***	0.4***	0.4	1.1	0.0**
*Diogoa zenkeri*	0.7	0.9	0.6	1.2	1.0	1.2	0.0	0.2*	0.6	0.6	0.5	3.3	1.5	0.5	0.5	0.0***	0.5	0.0	1.1
*Engomegona gordonii*	0.7	0.2	0.0	1.2	1.2	1.2	5.4	0.2*	34.5	0.4*	0.8	0.2	0.8	0.4	1.4	0.3***	0.5	1.4	0.0
*Scorodocarpus borneensis*	1.4	0.5	1.1	0.6	0.0	0.7	0.7	0.5	0.0	1.0	0.8	0.7	0.8	0.7	0.9	0.0***	0.0*	0.5	0.6
*Strombosia pustulata*	1.1	0.8	0.7	0.0	0.5	1.0	0.9	0.1**	0.5	1.1	0.2**	1.3	0.5	0.7	1.2	0.0***	0.0	1.2	1.1
*Strombosiopsis tetrandra*	0.7	0.6	0.8	24.6	1.2	1.2	0.7	0.5	0.2	0.9	0.1	0.7	0.8	0.7	0.2	0.1***	0.5	0.0	17.6
*Tetrastylidium peruvianum*	0.6	1.0	0.8	0.0	1.2	1.2	4.6	0.5	0.4	0.5	35.0	3.6*	0.3	0.7	0.7	0.0***	0.5	11.0	0.0
*Coula edulis*	0.7	22.6	0.8	0.0	1.2	12.5	1.1	21.1	10.9	1.4	3.4	3.3	0.5	0.6	0.0**	0.8	0.5	1.2	1.2
*Minquartia guianensis*	0.7	0.1	0.1	50.0	1.2	1.2	0.4	1.1	33.2	0.6	0.5	2.4	0.1	0.4	3.3**	0.0***	1.4	1.2	0.0
*Octoknema affinis*	0.7	9.4*	0.6	2.3	0.6	14.2	1.1	0.1**	1.1	0.7	0.7	1.1	0.6	0.9	1.0	0.0***	1.0	0.0*	0.5
*Anacolosa papuana*	0.0	0.5	0.2	0.9	0.0	0.0*	0.3	–	0.3	2.9	50.0	0.6	1.4	0.4	0.8	0.0***	1.1	1.2	0.2
*Anacolosa pervilleana*	0.7	0.9	1.3	0.0	5.5	1.2	0.6	–	0.3	0.4***	0.5	1.1	0.4	0.4	0.9	0.0***	0.0*	1.2	1.0
*Aptandra tubicina*	0.0	0.8	0.0*	1.2	0.4	0.8	0.0	–	0.4	0.9	0.9	0.0	0.9	0.6*	0.6	0.0***	0.0**	0.5	0.0*
*Cathedra acuminata*	2.0	0.6*	1.2	0.0	1.2	1.2	0.1	–	0.5	0.6*	0.9	1.2	0.6	0.8	0.3***	0.0***	3.2**	13.1	0.7
*Harmandia mekongensis*	0.5	0.6***	1.1	1.0	0.0	0.0*	0.3	–	0.6	0.3***	0.8**	1.7	0.3	0.7	20.4**	0.3***	0.4	0.6	0.7
*Phanerodiscus capuronii*	0.7	0.9	0.6	0.0	0.4	21.0	1.3	–	1.2	0.6**	0.5***	0.4	0.5	0.7	0.4**	0.3***	0.5	1.0	0.6
*Curupira tefeensis*	1.0	0.3*	0.7	0.0	1.2	11.0	0.7	–	0.8	1.2	0.4***	0.4	1.6	0.9	0.5	0.1***	1.8*	1.0	0.8
*Malania oleifera*	0.8	0.5**	0.6	0.2	0.9	0.5	0.9	–	0.0*	0.6***	3.2	0.0	0.8	0.6**	2.1	0.5***	0.5	0.4	0.5
*Ximenia americana*	0.7	0.5	1.0	0.0	1.0	1.2	0.3	–	0.1	0.8	0.7	1.9	0.6	1.3	1.0	0.1**	3.0***	1.2	31.5
*Dulacia candida*	1.6	0.6	0.5	1.9	0.0	–	0.0	–	1.1	0.8	0.7	1.9*	1.5	1.0	0.8	0.7	0.5	0.0	1.2
*Olax imbricata*	0.7	1.0	0.1	0.7	1.0	–	4.4	–	0.6	1.1	0.6	1.6	1.7	25.1	0.3	0.0**	3.2	14.5	0.0
*Olax scandens*	0.7	1.7	0.7	0.0	11.0	–	0.0	–	0.2	3.8	0.9	1.2	2.7*	0.6	0.7	0.3	2.0*	1.2	0.0
*Schoephia arenaria*	1.2	0.9	1.2	1.4	6.0	1.2	1.2	–	0.2	0.3	0.0	1.6	1.3	0.5	0.0	0.0***	0.0	1.0	1.2
*Schoephia schreberi*	4.2	0.3	0.8	1.2	0.0	1.2	0.3	–	0.2	0.4	0.0**	2.2*	1.1	0.3	0.4	0.3***	0.4	11.4	1.2
*Misodendrum brachystachyum*	1.1	0.3**	0.7	35.1	0.1	1.0	1.4	–	0.6**	0.7***	0.0**	0.0**	0.6	0.9	0.0	31.7	0.6	0.8	1.1
*Loranthus europaeus*	0.3*	0.4***	0.5*	0.1***	1.6	0.0*	0.2*	–	0.6***	0.3***	0.6***	0.0**	0.2***	0.7***	0.0***	33.5	0.5	0.8	0.7
*Viscum trachycarpum*	3.2***	0.0***	0.0**	0.7	12.6	–	9.4*	–	0.6***	0.7***	0.3***	33.3	0.7*	0.2***	21.3***	–	2.3***	0.5*	0.9
*Nanodea muscosa*	0.5	0.5**	0.6	0.3*	50.0*	–	0.4*	–	0.6	0.8	0.9	0.0**	0.7	0.2**	0.7*	0.4	1.2	1.1	0.6
*Thesium decaryanum*	0.7*	0.8*	0.7	1.1	4.5*	–	1.1	–	1.6	0.6	0.8*	0.0*	0.9	1.0	1.0	0.0***	0.0**	1.0	1.1

In addition to Erythropalaceae, Octoknemaceae and some Aptandraceae showed significantly relaxed selection of the combined group of *ndh* genes (Table 3). However, when investigating the 11 *ndh* genes separately for the 13 species that possess these genes, significantly relaxed selection was observed in only 12 % and this was confined to members of Erythropalaceae and Aptandraceae (Supplementary Data Tables S4 and S5). *Erythropalum scandens* had the highest number of genes (six) with significantly relaxed selection, and *ndhF* was the gene most often under significantly relaxed selection (in six species).

### Presence of infA in the nuclear genome

A transcript containing an *infA*-like sequence was recovered from the *Viscum album* transcriptome assembly, as well as from *Santalum album* and *Taxillus chinensis*. The transcript has a 498-bp-long open reading frame in *Viscum*, 474 bp in *Santalum* and 858 bp in *Taxillus*, i.e. more than twice the length of the gene when present in the plastome of species of Santalales and outgroup taxa (234 bp). The extended sequences in *Viscum*, *Santalum* and *Taxillus* were all found to correspond to N-terminal presequences predicted by TargetP-2.0 to be thylakoid luminal transit peptides with a predicted cleavage site between amino acid residues 63 and 64 (*Viscum*), 70 and 71 (*Santalum*), and 74 and 75 (*Taxillus*) (Fig. 3).

## DISCUSSION

### Phylogenetic relationships and evolution of parasitism

The clades recovered here at the rank of family (Fig. 3) correspond to the family delineation of Nickrent *et al.* (2010, 2019) and Su *et al.* (2015), and the topologies within these clades are also consistent. The current study differs from this previous work by utilizing significantly more sequence data that provided better support for several deep splits in the phylogeny. However, some relationships remain weakly supported.

The combined phylogenetic analyses conducted here strongly support a sister group relationship between Strombosiaceae and Erythropalaceae, and this clade is strongly supported as sister to the rest of the Santalales (Fig. 3; Supplementary Data Fig. S3). In contrast, most previous phylogenetic analyses have placed either Erythropalaceae or Strombosiaceae as sister to the remaining Santalales (e.g. Su *et al*., 2015; Nickrent *et al.*, 2019; Nickrent, 2020) (Fig. 1). However, Nickrent *et al*. (2019) used *Erythropalum* to root the phylogeny, which affected the topology. In Su *et al.* (2015), combined analysis of seven genes from all genomic compartments very weakly supported the consecutive branching order Strombosiaceae, Erythropalaceae and the remaining order, but their analysis excluding the plastid genes weakly supported a monophyletic Strombosiaceae + Erythropalaceae sister to the rest of the order. A conflicting phylogenetic signal from nuclear versus plastid data was also detected in the present study. Analyses of the plastid data give results congruent with combined analyses, whereas analyses of nuclear data recover a variably supported clade with Erythropalaceae, Strombosiaceae, Coulaceae and Octoknemaceae (Figs S1–S3). In previous studies the phylogenetic position of Octoknemaceae, including only the genus *Octoknema*, has been ambiguous (see Fig. 1). In the seven-gene analysis of Su *et al.* (2015), *Octoknema* was found in an unresolved position in relation to two clades including all families other than Erythropalaceae and Strombosiaceae. In Nickrent *et al.* (2019), *Octoknema* was placed as a weakly supported sister group to a clade of confirmed parasitic families, but after adding additional sequence data, a sister group relationship to Strombosiaceae was suggested instead (Nickrent, 2020). In the present study, *Octoknema* is consistently placed as the sister group to Coulaceae (Figs 3 and S1–S3), but support is mostly weak. At the same time, the conflicting position of the Coulaceae + *Octoknema* clade recovered by nuclear versus plastid data indicates that further data will be needed to shed light on the relationships. Irrespective of the placement of *Octoknema* and Coulaceae within a clade including Erythropalaceae and Strombosiaceae or as sister to the confirmed parasites and Aptandraceae, the position is consistent with the contention that *Octoknema* is non-parasitic.

Despite relatively weak support for a clade including Aptandraceae, Ximeniaceae, Olacaceae and the remaining parasites in the combined maximum likelihood analysis (UB 76, but 1 pp from Bayesian inference), all analyses recover these taxa in a monophyletic group (Fig. 3; Supplementary Data Figs S1–S3). However, internal topological differences can be observed both from analyses of different data partitions and to some extent from maximum likelihood versus Bayesian inference. This is consistent with previous observations demonstrating incongruence between data signal from different genomic compartments, but also by the choice of phylogenetic method, primarily parsimony versus maximum likelihood or Bayesian inference (Su *et al.*, 2015). The topological differences observed here have only weak support from the maximum likelihood analyses with no relationship between any of the four groups having a higher support than UB 76 %. However, Bayesian inference tends to assign numerically higher posterior probabilities to the resolved clades (Cummings *et al*., 2003), thus inflating conflict between the nuclear and plastid data partitions (Fig. S3). Since the amount of nuclear data used in the current study is limited, additional nuclear data are needed for further exploring the potential intergenomic data conflict and ultimately for resolving relationships within the clade. Additional nuclear data may also help shed light on the biological reason(s) for observed data partition incongruence. Independent lineage sorting, chloroplast capture and introgression are well-known causes of incongruence, but with the current data for Santalales, only speculations are possible.

Topological ambiguity of the Santalales phylogeny obviously affects interpretation of the evolution of parasitism. Tentatively assuming that the phylogenetic hypothesis derived from maximum likelihood analysis of the combined data set (Fig. 3) is correct, parasitism probably evolved once, either in the ancestor to the clade including all confirmed parasitic families, or – if Aptandraceae is parasitic – in the common ancestor to the larger clade including both groups. Although haustoria have never been reported from any species of Aptandraceae, this cannot be used to infer that they are not parasitic (Nickrent, 2020), and parasitism has been proposed based on the position of the family in earlier phylogenetic analyses (Malécot *et al.*, 2004). However, the phylogenetic position with Aptandraceae sister to the confirmed parasites is equally consistent with a non-parasitic status of Aptandraceae as suggested by Kuijt and Hansen (2015). Any alternative position of Aptandraceae within the clade of confirmed parasites will complicate the scenario for evolution of parasitism, but only if the family is non-parasitic. Either parasitism would have evolved twice or Aptandraceae would have reverted to an autotrophic habit. The latter scenario has never been demonstrated in any other clade of parasitic plants (Nickrent, 2020).

The non-parasitic status of Erythropalaceae, Strombosiaceae and Coulaceae has been inferred by the absence of haustoria in those few species investigated (Kuijt and Hansen, 2015; Nickrent, 2020). As above, this does not necessarily imply that all members of the families are non-parasitic, but currently no evidence exists for parasitism in the groups. The same applies to *Octoknema*, where no haustoria have been reported (Gosline and Malécot, 2011). Potential parasitism has been suggested for this family, but entirely based on a previously inferred phylogenetic position among parasitic taxa (Nickrent *et al.*, 2010). However, both the current phylogenetic hypotheses and the relationships suggested in Nickrent (2020) are consistent with a non-parasitic status.

Further ambiguity about the status of parasitism even applies to Olacaceae and Ximeniaceae. Although we have scored both families as parasitic here, as discussed in Nickrent (2020), haustoria have been documented only in *Olax*, *Ptychopetalum* and *Ximenia*. Thus, tracing the origin(s) of parasitism in Santalales precisely still needs both a stronger supported phylogenetic framework and greater knowledge about parasitism or non-parasitism in members of the families now assumed to be non-parasitic or root parasitic.

### Gene losses

In concordance with previous studies of Santalales plastomes (Petersen *et al.*, 2015; Zhang *et al.*, 2015; Su and Hu, 2016; G.-S. Yang *et al.*, 2017; Li *et al.*, 2017; Shin and Lee, 2018; Zhu *et al.*, 2018; Guo and Ruan, 2019*a*, *b*; Guo *et al.*, 2019, 2020, 2021*a*, *b*; Jiang *et al.*, 2019; S.-S. Liu *et al.*, 2019; Schelkunov *et al.*, 2019; Su *et al.*, 2019, 2021; Y. Yang and He, 2019; Yu *et al.*, 2019; Chen *et al.*, 2020; D. Yang *et al*., 2020; Ceriotti *et al.*, 2021; Nickrent *et al.*, 2021; X. Liu *et al*., 2021; Al-Juhani *et al.*, 2022; Darshetkar *et al*., 2023; Tang *et al.*, 2024) as well as with investigations of other lineages of hemiparasites and photosynthetic mycoheterotrophs in general (Wicke *et al.*, 2016; Frailey *et al.*, 2018; Wicke and Naumann, 2018; Kim *et al.*, 2020), hemiparasitic root parasites in the Santalales have undergone relatively few gene losses (Fig. 3). A notable exception is the extensive gene loss reported in Ximeniaceae by Guo *et al.* (2020), where not only the *ndh* genes were reported as lost, but also *matK*, *psaC*, *psbA,I,K*, *rpl32*, *rps15*, *rps16*, *ycf1* as well as eight tRNA genes. This conclusion was based on the 125 050-bp-long accession MG799332 of *Malania oleifera*, originally published in Liu *et al.* (2019), and the unverified accession MN414175 of *Ximenia americana*, originally published in Chen *et al.* (2020). Though gene loss was reported in Guo *et al.* (2020) for *Ximenia*, the original publication by Chen *et al.* (2020) reported the presence of all these genes, but they are not annotated in the available GenBank accession. However, local blastN searches (as implemented in Geneious Prime v.2019.0.4) using our new assembly of *Ximenia americana* confirm that all genes are present in accession MN414175, as originally reported in Chen *et al.* (2020). Therefore, the loss reported by Guo *et al.* (2020) for accession MN414175 of *Ximenia americana* is probably the result of annotation problems. Although we were unable to locate genes reported as missing in Guo *et al.* (2020) in accession MG799332 of *Malania oleifera*, they are present in the much longer accession MK764537 (158 163 bp long) published in Yang and He (2019), which we included in the current study because it shows a higher degree of similarity with that of *Ximenia americana* (both in our new assembly and in accession MN414175).

The phylogenetic relationships obtained here support a single functional loss of the 11 *ndh* genes from the plastome in the common ancestor to the group formed by confirmed parasites and the family Aptandraceae (Fig. 3; Supplementary Data Fig. S3). If Aptandraceae is parasitic (and Octoknemaceae non-parasitic), the loss of the plastid *ndh* genes would coincide with the transition to a parasitic lifestyle, further supporting previous claims of correlation between some degree of heterotrophism (haustorial parasitism and mycoheterotrophy) and loss of plastid *ndh* genes (Wicke *et al.*, 2011; Graham *et al.*, 2017; Wicke and Naumann, 2018; Chen *et al.*, 2020). However, if Aptandraceae is not parasitic, loss of *ndh* genes pre-dates evolution. Although loss of the plastid *ndh* genes is common to almost all parasitic and mycoheterotrophic lineages, it has been reported in an increasing number of non-parasitic lineages as well (e.g. Martín and Sabater, 2010; Blazier *et al.*, 2011; Sun *et al.*, 2018; Nevill *et al.*, 2019; Morais da Silva *et al.*, 2021). The plastid *ndh* genes code for some of the subunits of the NDH complex, involved in the minor pathway of photosystem I cyclic electron transport; however, an alternative major pathway is mediated by the entirely nuclear encoded PGR5/PGRL1 protein complex (Shikanai, 2020; Ma *et al.*, 2021). The partial redundancy of both pathways may explain the recurrent loss of plastid *ndh* genes even in a number of autotrophs (see e.g. Strand *et al.*, 2019). The importance of the NDH-dependent pathway has been associated with numerous factors of environmental stress, such as low, high or fluctuating light conditions, low, high or fluctuating temperatures, drought, and phosphorus deficiency. Although oxidative stress appears to be a key factor, the complete physiological and possibly dual functions of the NDH complex remain unclear (for recent reviews see Yamori and Shikanai, 2016; Strand *et al.*, 2019; Shikanai, 2020; Ma *et al.*, 2021). Repeated loss of *ndh* genes and probably the entire NDH complex in several autotrophs may suggest that a similar loss in parasites and other heterotrophs is not related to their lifestyle per se. If loss of *ndh* genes in Santalales pre-dates evolution of parasitism, it could rather be hypothesized that the loss was a driving force towards a heterotrophic lifestyle. However, this is neither the case in Orobanchaceae, where loss of *ndh* genes appears to be correlated with the transition to hemiparasitism (Zhang *et al.*, 2020), nor in Krameriaceae, where hemiparasitism has evolved without functional loss of *ndh* genes (Banerjee *et al*., 2022).

Among angiosperms, the *infA* gene, coding for translation initiation factor A, has been lost repeatedly from the plastome (Millen *et al.*, 2001; Daniell *et al.*, 2016). Within Santalales, Chen *et al.* (2020) reported the loss of *infA* in all investigated species (both root- and stem-feeding parasites) except *Erythropalum scandens* and ‘early diverging root-(hemi)parasites’ (*Malania oleifera*, *Ximenia americana*, *Schoepfia fragrans* and *S. jasminiodora*). Although our findings concur with Chen *et al.* (2020) regarding the presence of *infA* in Ximeniaceae and *Schoepfia* spp., we found it in the stem parasitic *Misodendrum brachystachyum* as well but not in Olacaceae. In Loranthaceae, the functional loss of *infA* in *Loranthus* was reported previously (Nickrent *et al*., 2021). Similar functional losses apply to most investigated genera of Loranthaceae, but intact *infA* genes were found in *Nuytsia* and *Moquiniella* (Nickrent *et al*., 2021; Tang *et al.*, 2024). Phylogenetic evidence suggests that *infA* was lost more than once from the plastome in Loranthaceae (Tang *et al.*, 2024). On the general assumption that acquisition after loss is unlikely, loss of *infA* has occurred repeatedly within Santalales, and no clear parallel can be drawn between loss and a parasitic lifestyle.

**Fig. 4.**
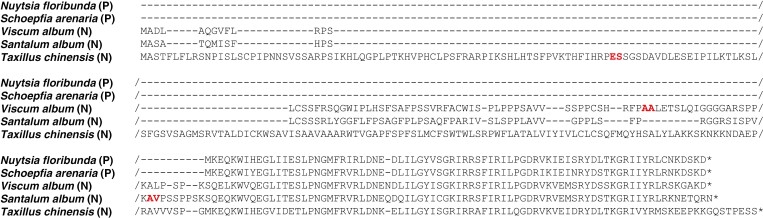
Comparison between plastid (P) and putative nuclear (N) *infA* amino acid sequences. Predicted cleavage sites are in bold/red.

In contrast to the completely lost *ndh* genes, the presence of homologous genes in the nuclear genome has previously been demonstrated for *infA* in *Arabidopsis thaliana* (Brassicaceae), *Glycine max* (Fabaceae), *Solanum lycopersicum* (Solanaceae) and *Mesembryanthemum crystallinum* (Aizoaceae) (Millen *et al.*, 2001). They all have nuclear-encoded *infA* genes with transit peptide presequences comparable to those of *Viscum album*, *Santalum album* and *Taxillus chinensis*, albeit all are highly dissimilar and probably reflect independent events of transfer from the plastome to the nuclear genome. The pattern of *infA* presence/absence in Santalales (Fig. 2; Nickrent *et al.*, 2021; Su *et al.*, 2021) suggests that the gene was ancestrally present, but subsequently functionally or completely lost from the plastome at least three times: in Olacaceae, in Loranthaceae (where it might have been lost more than once) and in a clade corresponding to Santalaceae *s.l*. In the current study the last clade is represented by Nanodeaceae, Thesiaceae and Viscaceae, but also Santalaceae *s.s*., Amphorogynaceae, Cervantesiaceae and Comandraceae (Nickrent, 2020). These losses are consistent with the data from previously published complete plastomes of the Santalales, including sequences from families being underrepresented or not represented at all in the current analysis (Petersen *et al.*, 2015; Zhang *et al.*, 2015; Su and Hu, 2016; G.-S. Yang *et al.*, 2017; Li *et al.*, 2017; Shin and Lee, 2018; Zhu *et al.*, 2018; Guo and Ruan, 2019*a*, *b*; Guo *et al.*, 2019, 2020, 2021*a*, *b*; Jiang *et al.*, 2019; S.-S. Liu *et al.*, 2019; Schelkunov *et al.*, 2019; Su *et al.*, 2019, 2021; Y. Yang and He, 2019; Yu *et al.*, 2019; Chen *et al.*, 2020; D. Yang *et al*., 2020; Ceriotti *et al.*, 2021; Nickrent *et al.*, 2021; X. Liu *et al*., 2021; Al-Juhani *et al.*, 2022; Darshetkar *et al*., 2023; Tang *et al.*, 2024). The presence of potentially functional nuclear *infA* genes in *Viscum album*, *Santalum album* and *Taxillus chinensis* suggests at least one event of intracellular gene transfer, but more independent events cannot be ruled out. The higher similarity of the transit peptide presequences of *Viscum* and *Santalum* (see Fig. 4), both members of the Santalaceae *s.l*. clade, makes a common origin for these sequences likely, but nuclear data from more representatives from the clade would be desirable. After the establishment of a functional nuclear gene, the plastid gene copy is expected to become superfluous and experience relaxed selection, pseudogenization and loss. Indeed, pseudogenes and complete losses are observed in three clades. If intracellular transfer and establishment of a functional nuclear gene occurred only once in a common ancestor to the three clades, it could be expected that Schoepfiaceae and Misodendraceae, which are derived from the same ancestor and still possess plastome *infA* genes, would show signs of relaxed selection. However, this is not the case and may indicate a scenario of several independent transfer events in clades lacking functional plastome *infA* genes.

### Patterns of relaxed selection

The tests for relaxed and intensified selection provide a mixed picture with only a few clades showing a consistent pattern of relaxation of selection for individual genes or functional groups of genes. At the same time, comparison with data from similar studies of the Santalales or other groups of heterotrophic plants are not straightforward because of differences in taxon sampling, differences in how genes are treated (as individual units or functional groups) and differences in how the tests are performed. Here we surprisingly detected issues with convergence of results among individual runs using RELAX. As a random-effects model, RELAX may be sensitive to small sets of test and reference branches (S. Pond, pers. comm.). Adjusting the settings as described above improved convergence, but a few repeated runs still gave significantly different results. To our knowledge this has not been reported in other studies, which usually report a single run.

Overall there is no clear difference in patterns of relaxation between non-parasitic and parasitic taxa. Given that all parasitic species included here are hemiparasites that might not be expected, although Petersen *et al.* (2015) did find relaxed selection of photosynthesis genes based on a Santalales sampling limited to *Viscum* and *Osyris* (Santalaceae). In Orobanchaceae relaxed selection has also been reported for some hemiparasitic taxa (Wicke *et al.*, 2016; Li *et al.*, 2021). Among the hemiparasitic taxa in the current study, the three stem parasites, *Misodendrum*, *Loranthus* and *Viscum*, show a tendency towards a higher degree of relaxed selection, in particular in photosynthesis genes compared to the root parasitic taxa. The same observation was made by Chen *et al.* (2020) and may be explained by increased acquisition of host carbon and a reduced need for photosynthesis in stem parasites compared to root parasites (Těšitel, 2016).

Among the non-parasitic clades, Erythropalaceae stands out as having considerably more genes and gene groups showing significantly relaxed selection compared to Strombosiaceae and Coulaceae (and Octoknemaceae, although the parasitic status is not known). In contrast to the three stem parasites, relaxed selection does not involve all photosynthesis-related genes. However, significantly relaxed selection can be observed for the combined group of *ndh* genes in Erythropalaceae, Octoknemaceae and some Strombosiaceae. Testing for relaxed selection in the individual *ndh* genes showed only a few significant changes, most involving *ndhF*. This might suggest that *ndhF* could be the first gene to be affected by relaxed selection, but with the longest coding region among the 11 *ndh* genes, it may be that it is easier to detect significant changes for *ndhF*. The effect of gene length can be seen for *Octoknema affinis*, where significant relaxed selection was observed for the combined group of *ndh* genes, but none of the individual gene tests gave significant results.

Although relaxed selection of the *ndh* gene complement is not observed for all non-parasites, Coulaceae and some Strombosiaceae being exceptions, the results are consistent with a hypothesis that relaxed selection of the *ndh* genes could lead to their loss in the hemiparasitic clade (including Aptandraceae). In a comparable study of a clade of orchids, Neottieae, including both autotrophs, and partially and fully heterotrophic species, no significantly relaxed selection of *ndh* genes was detected in the autotrophic species prior to complete or functional loss of the genes in the heterotrophs (Lin *et al.*, 2017; Lallemand *et al.*, 2019). However, relaxed selection may apply to the entire clade of orchids compared to their monocot relatives.

In addition to the loss of *ndh* genes, three independent losses of *infA* were observed. As for the *ndh* loss, none of these losses are pre-dated by relaxed selection of *infA*. Since *infA* was probably transferred to the nuclear genome, rather than lost completely, relaxed selection would only be expected to apply to a remaining plastid gene copy following a functionally successful transfer event. Thus, the lack of relaxed selection of *infA* in all but one of the included representatives of Santalales may support transfer as multiple independent events.

### IR size and boundary shifts

Whereas little variation in IR size and boundaries is found among the confirmed non-parasitic species and *Octoknema affinis*, expansion and contraction of the IR have occurred within the clade including confirmed parasites and Aptandraceae (Fig. 2). Among non-parasites and *Octoknema*, the size of the IR ranges between 26 046 bp (*Heisteria densifrons*, Erythropalaceae) and 26 394 bp (*Erythropalum scandens*), with the IR–LSC (Long Single Copy) junction placed between *trnN-GUU* and *ycf1*, and with the IR–SSC junction between *rpl2* and *rps19* (Fig. 2), which seems to be the ancestral state in angiosperms (Zhu *et al.*, 2016). In the clade including parasites and Aptandraceae, the IR ranges between 12 426 bp (*Schoepfia schreberi*) and 35 256 bp (*Malania oleifera*), exhibiting major border shifts and inclusion or exclusion of several genes. The greatest deviations in size, border and gene content of the IR are observed in some root parasitic lineages (Ximeniaceae, Olacaceae, Schoepfiaceae) but not in the stem parasites included here (*Viscum*, *Loranthus*, *Misodendrum*), where dependency on parasitism might possibly be more strongly manifested. Despite our limited sample size of stem parasites, additional support for this observation can be found in Chen *et al.* (2020) where a much denser sampling of stem parasites revealed only minor differences in IR size and boundaries.

The major expansions of the IR in Ximeniaceae and Olacaceae appear to result from two independent events: a possible shared ancestral shift of the IR–SSC border and a shift of the IR–LSC border in Olacaceae, only. Whereas both these events have resulted in a larger IR gene content, a shift of the IR–LSC boundary in Schoepfiaceae caused a major IR size reduction and several genes are no longer included in the IR region. Within Aptandraceae both contractions and expansions, mostly at the IR–SSC boundary, are also observed but far less pronounced than in the three confirmed stem parasitic families.

Among the representatives of Santalales included here, variation in IR size is largely explained by boundary shifts and only to a minor extent by loss of genes (e.g. *ndhB*). Similar instability of IR boundaries, mostly leading to IR expansion, has been observed in hemiparasitic Orobanchaceae (Frailey *et al.*, 2018; Li *et al.*, 2021). Orchidaceae also shows IR instability prior to complete loss of one copy in a few fully mycoheterotrophic taxa (Kim *et al.*, 2020). Complete loss of one IR copy was even observed in the hemiparasites *Cassytha* (Lauraceae) and *Cuscuta* subgenus *Cuscuta* (Convolvulaceae) (Wu *et al.*, 2017; Banerjee and Stefanović, 2020; Yu *et al*., 2023). Owing to a copy-dependent repair mechanism, genes located within the IR regions have lower synonymous substitution rates compared to genes in the single-copy regions (Zhu *et al.*, 2016). Thus, expanding IR boundaries can lead to increased conservation and boundary contraction to decreased conservation of affected genes. In Santalales this probably explains the retention of some *ndh* pseudogenes in Ximeniaceae and Olacaceae, which are otherwise completely lost. Whether IR instability can be linked to a heterotrophic lifestyle is not clear, and both instability and loss are reported also from autotrophic taxa (see Zhu *et al.*, 2016). Major IR boundary shifts in Santalales are only observed in confirmed parasitic clades, but are not a characteristic of all. Some shifts are even observed in Aptandraceae, but the uncertain parasitic status precludes any firm conclusions. However, IR instability is correlated with the functional loss of *ndh* genes in the ancestor to Aptandraceae plus confirmed parasites.

## CONCLUSION

With the addition of 29 new plastome assemblies, we provide a framework for the study of plastome configuration and evolution in non-parasitic and hemiparasitic Santalales. Phylogenetic patterns indicate a single loss of the plastid *ndh* genes, and signs of relaxed selection of these genes can be observed prior to the functional loss. Loss of *ndh* coincides with an apparent instability of the IR borders in the group formed by confirmed parasites and Aptandraceae. However, correlation with the transition to a parasitic lifestyle remains uncertain, as the parasitic status of Aptandraceae and Octoknemaceae is yet to be determined. Although loss of the *ndh* gene complex is one of the most common and unifying traits of plastomes from parasitic and mycoheterotrophic plants (Wicke *et al.*, 2011; Graham *et al.*, 2017; Wicke and Naumann, 2018; Chen *et al.*, 2020), similar losses are continuously being reported in autotrophic plants (e.g. Martín and Sabater, 2010; Blazier *et al.*, 2011; Sun *et al.*, 2018; Nevill *et al.*, 2019; Morais da Silva *et al.*, 2021). For these reasons, determining the parasitic status of Aptandraceae is therefore crucial for interpreting the potential correlation between parasitism and *ndh* loss in Santalales. The same applies to potential correlation between parasitism and plastome instability in general.

Additionally, we conclude that repeated loss of *infA* from the plastome is best explained by a pattern of (repeated) transfer to the nuclear genome rather than repeated functional loss of the gene. Although these transfer events have taken place in parasitic lineages, there is no evidence they are connected to parasitism.

The relatively few gene losses observed in hemiparasites is consistent with most previous findings, indicating that, except for the loss of the plastid encoded subunits of the NDH complex, the function of the plastid remains relatively unaltered in hemiparasites within Santalales, and that efficient photosynthesis is probably maintained and important.

In addition to uncertainty about parasitism in some lineages of Santalales, some of the deeper splits in the phylogeny lack solid support. Additional taxon sampling and molecular data (particularly from the nuclear genome) are desirable to further test phylogenetic hypotheses and potential data partition incongruence.

## SUPPLEMENTARY DATA

Supplementary data are available at *Annals of Botany* online and consist of the following.

Table S1: Origin of samples and information about reference sequences used for assembly. Table S2: Number of reads, average read depth (ARD) and GenBank accession numbers of new sequences. Table S3: Genes obtained in partially assembled plastomes. Table S4: Detailed results from five runs of RELAX showing relaxed or intensified selection in functional gene groups and individual genes. Table S5: Patterns of relaxed and intensified selection of individual *ndh* genes. Fig. S1. Phylogeny of Santalales based on ML analysis of plastome gene sequences. Node values are ultrafast bootstrap support. Fig. S2. Phylogeny of Santalales based on ML analysis of nrDNA sequences. Node values are ultrafast bootstrap support. Fig. S3. Bayesian majority rule consensus tree from analysis of the plastid/nuclear/combined alignment. Fig. S4. SplitsTree4 consensus network of the credible set of trees from Bayesian analysis of the plastid/nuclear/combined alignment.

mcae145_suppl_Supplementary_Figure_S1

mcae145_suppl_Supplementary_Figure_S2

mcae145_suppl_Supplementary_Figure_S3

mcae145_suppl_Supplementary_Figure_S4

mcae145_suppl_Supplementary_Table_S1

mcae145_suppl_Supplementary_Table_S2

mcae145_suppl_Supplementary_Table_S3

mcae145_suppl_Supplementary_Table_S4

mcae145_suppl_Supplementary_Table_S5
