# Supplementary material for: Plastome evolution in Santalales involves relaxed selection prior to loss of ndh genes and major boundary shifts of the inverted repeat
Source: Ann Bot. 2024 Aug 30;135(3):515–30. doi: 10.1093/aob/mcae145 (PMC11897430; doi:10.1093/aob/mcae145)
Supplement: mcae145_suppl_Supplementary_Table_S1 [file mcae145_suppl_supplementary_table_s1.docx]

**Table S1. Collection and sequence assembly information**

| **Species** | **Collector and no.** | **DNA accession no.** | **Voucher location^1^** | **Plastome reference^2^** | **nrDNA reference^3^** | **KAT-filtered^4^** |
| --- | --- | --- | --- | --- | --- | --- |
| *Brachynema ramiflorum* Benth. | C. A. Cid & al. 1482 | DLN 4378 | NY | *Erythropalum scandens*, NC036759.1 | MH390490.1, MH390532.1 | no |
| *Heisteria densifrons* Engl. | J. K. Munzinger, M. Pigmal, and O. Ponaj No. 497 | DLN 4232 | P | *Erythropalum scandens*, NC036759.1 | MH390491.1, MH390534.1 | no |
| *Maburea trinervis* Maas | R. Zagt s.n. | DLN 6011 | WAG | *Erythropalum scandens*, NC036759.1 | DQ790114.1, MH390533.1 | no |
| *Diogoa zenkeri* (Engl.) Exell & Medonça | J. J. Wieringa & Fl van Nek 3288 | DLN 3078 | WAG | *Erythropalum scandens*, NC036759.1 | MH390493.1, MH390535.1 | no |
| *Engomegoma gordonii* F.J.Breteler | B. Senterra no. 18-84 | DLN 4556 | ULB | *Erythropalum scandens*, NC036759.1 | DQ790110.1 | yes |
| *Scorodocarpus borneensis* (Baill.) Becc. | Julisasa T. Hadiah s.n. | Su103 | Bogor Botanical Garden | *Nicotiana tabacum*, Z00044.2 | MT968896.1 | no |
| *Strombosia pustulata* Oliv. | J. J. Wieringa & T. Nzabi No. 2781 | DLN 4054 | WAG | *Erythropalum scandens*, NC036759.1 | MH390494.1, MH390536.1 | no |
| *Strombosiopsis tetrandra* Engl. | J. J. Wieringa & Fl van Nek 3300 | DLN 4055 | WAG | *Erythropalum scandens*, NC036759.1 | MH390495.1, MH390537.1 | no |
| *Tetrastylidium peruvianum* Sleumer | Henk van der Werff & R. Vasquez 13875 | DLN 4205 | MO | *Erythropalum scandens*, NC036759.1 | MH390496.1, MH390538.1 | no |
| *Coula edulis* Baill. | J. J. Wieringa & Fl van Nek 3295 | DLN 3079 | WAG | *Erythropalum scandens*, NC036759.1 | MH390497, MH390539.1 | no |
| *Minquartia guianensis* Aubl. | D. L. Nickrent 2758 | DLN 2758 | BH | *Erythropalum scandens*, NC036759.1 | MH390497 | yes |
| *Octoknema affinis* Pierre | X. M. van der Burgt & J. Motoh 821 | DLN 6751 | WAG | *Erythropalum scandens*, NC036759.1 | DQ790117.1 | no |
| *Anacolosa papuana* Schellenb. | R. Regalado and M. Q. Sirikolo 692 | DLN 4247 | MO | *Erythropalum scandens*, NC036759.1 | DQ790104.1 | yes |
| *Anacolosa pervilleana* Baill. | Razafimandibison et al. 2218 | 2218 | S | *Erythropalum scandens*, NC036759.1 | DQ790104.1 | yes |
| *Aptandra tubicina* (Poepp.) Benth. ex Miers | H. van der Werff & R. Vasquez 13846 | DLN 4202 | MO | *Erythropalum scandens*, NC036759.1 | DQ790105.1 | yes |
| *Cathedra acuminata* (Benth.) Miers | J. A. Ratter et al. 6782 | DLN 4244 | MO | *Erythropalum scandens*, NC036759.1 | FJ848847.1 | yes |
| *Harmandia mekongensis* Baill. | Koizumi 1411 | DLN 5597 | KYO | *Erythropalum scandens*, NC036759.1 | FJ848849.1 | yes |
| *Phanerodiscus capuronii* Malécot, G.E.Schatz & Bosser | G. E. Schatz, W. D. Stevens and J. P. Rakotomazaza 3439 | DLN 4204 | MO | *Erythropalum scandens*, NC036759.1 | MH390502.1, MH390544.1 | no |
| *Curupira tefeensis* Black | C. Clement s.n. | DLN 4988 | BH | *Malania oleifera*, MK764537.1 | MH390498.1, MH390540.1 | no |
| *Malania oleifera* Chun & S.K.Lee | Caoming 0340 | DLN 4158 | P | n.a. | MH390499.1, MH390541.1 | no |
| *Ximenia americana* L. | M.A. Caraballo-Ortiz | 3203 | PAC | *Malania oleifera*, MK764537.1 | MH390500.1, MH390542.1 | yes |
| *Dulacia candida* (Poepp.) Kuntze | D. Niel et al. 10956 | 10956 | GB | *Malania oleifera*, MK764537.1 | DQ790109.1 | yes |
| *Olax imbricata* Roxb. | Y,-H. Tseng and H.-J. Su | Su086 | TAIF | *Nicotiana tabacum*, Z00044.2 | MT968896.1 | no |
| *Olax scandens* Roxb. | P. J. Grote s. n. | DLN 4894 | BH | *Malania oleifera*, MK764537.1 | MH390501.1, MH390543.1 | no |
| *Schoepfia arenaria* Britton | M.A. Caraballo-Ortiz | 3035 | PAC | *Schoepfia jasminodora*, NC034228 | MH390505.1, MH390546.1 | yes |
| *Schoepfia schreberi* G.F.Gmel | W. Forstreuter FW9132 | DLN 6013 | MB | *Schoepfia jasminodora*, NC034228 | MH390505.1, MH390546.1 | no |
| *Misodendrum brachystachyum* DC | G. Amico 132 | DLN 4588 | BCRU | *Malania oleifera*, MK764537.1 | MH390503.1, MH390547.1 | no |
| *Loranthus europaeus* Jacq. | Carlos Reif s.n. | C3127 | C | n.a. | EU544331.1, EU544380.1 | no |
| *Thesium decaryanum* Cavaco & Keraudren | Razafimandibison et al. 2075 | 2075 | S | *Viscum minimum*, NC027829 | MH390512.1, MH390553.1 | yes |
| *Nanodea muscosa* C.F.Gaertn. | R. Lewis RL1824 (L.4221483) | RL1824 | L | *Viscum minimum*, NC027829 | MH390524.1, MH390566.1 | yes |
| *Viscum trachycarpum* Baker | Razafimandibison et al. 2249 | 2249 | S | *Viscum minimum*, NC027829 | MH390524.1, AF389275.1 | yes |

1. Voucher locations are specified using herbarium acronyms
2. Reference sequence used for k-mer analysis
3. Reference sequence(s) used for k-mer analysis
4. For samples with high numbers of sequence reads (Table S2), KAT-filtering, based on plastome K-mer analysis, was used to reduce the number of off-target sequence reads.
