# Supplementary material for: Plastome evolution in Santalales involves relaxed selection prior to loss of ndh genes and major boundary shifts of the inverted repeat
Source: Ann Bot. 2024 Aug 30;135(3):515–30. doi: 10.1093/aob/mcae145 (PMC11897430; doi:10.1093/aob/mcae145)
Supplement: mcae145_suppl_Supplementary_Table_S2 [file mcae145_suppl_supplementary_table_s2.docx]

**Table S2. Number of trimmed reads, average read depth (ARD) and GenBank accession numbers of new sequences**

| **Species** | **Trimmed reads** | **ARD plastome** | **ARD nrDNA** | **GenBank no.**  **Plastome** | **nrDNA** |
| --- | --- | --- | --- | --- | --- |
| *Anacolosa papuana* | 84,027,058 | 3153.9 | 10,556.7 | OR678211 | OR722490 |
| *Anacolosa pervilleana* | 72,875,816 | 3,610 | 6,631.7 | OR712469 | OR722491 |
| *Aptandra tubicina* | 106,841,518 | 801.1 | 7,803.3 | OR712470 | OR722492 |
| *Brachynema ramiflorum** | 3,697,838 | 57.2 | 512.1 | OR933668-OR933682 | OR722493 |
| *Cathedra acuminata* | 98,090,236 | 4,186.1 | 9,667 | OR712471 | OR722494 |
| *Coula edulis** | 4,975,143 | 63 | 557.3 | OR902600-OR902602 | OR722495 |
| *Curupira tefeensis** | 8,397,462 | 153.6 | 2,621.1 | OR933639-OR933643 | OR722496 |
| *Diogoa zenkeri* | 12,932,043 | 164.4 | n.a. | OR712472 | n.a. |
| *Dulacia candida* | 70,621,286 | 588.2 | 3,257 | OR712473 | OR722497 |
| *Engomegoma gordonii* | 71,221,190 | 274.9 | 3,200.4 | OR712474 | OR722498 |
| *Harmandia mekongensis* | 80,986,346 | 420.2 | 1,081.8 | OR712475 | OR722499 |
| *Heisteria densifrons** | 10,255,950 | 154.8 | 2,362.4 | OR933649-OR933655 | OR722500 |
| *Loranthus europaeus* | 31,826,976 | n.a. | 661.9 | n.a. | OR722501 |
| *Maburea trinervis** | 7,904,155 | 101.4 | 430.9 | OR933644-OR933648 | OR722502 |
| *Malania oleifera* | 12,959,702 | n.a. | 2,985.7 | n.a. | OR722503 |
| *Minquartia guianensis* | 117,436,038 | 3,147 | 12,134.2 | OR712476 | OR722504 |
| *Misodendrum brachystachyum* | 13,741,748 | 1876.0 | 2,501.9 | OR712477 | OR722505 |
| *Nanodea muscosa* | 55,240,120 | 2147.3 | 3,716.6 | OR712478 | OR722506 |
| *Octoknema affinis* | 6,182,572 | 201.5 | 1,431.3 | OR712479 | OR722507 |
| *Olax imbricata* | 4,483,152 | 1,160.8 | 1,592.9 | KX816863 | OR676936 |
| *Olax scandens** | 13,196,312 | 721.6 | 5,880.4 | OR915050-OR915052 | OR722508 |
| *Phanerodiscus capuronii** | 4,345,383 | 138.1 | 528.4 | OR933656-OR933667 | OR722509 |
| *Schoepfia arenaria* | 15,538,280 | 631.9 | 5,950.3 | OR712480 | OR722510 |
| *Schoepfia schreberi* | 6,732,799 | 616.8 | 3,322 | OR712481 | OR722511 |
| *Scorodocarpus borneensis* | 4,888,874 | 106.2 | 823.7 | OR684446 | OR676937, OR676938 |
| *Strombosia pustulata* | 11,646,684 | 244.5 | 3,637.4 | OR712482 | OR722512 |
| *Strombosiopsis tetrandra* | 8,573,748 | 101.1 | 862.8 | OR712483 | OR722513 |
| *Tetrastylidium peruvianum** | 5,372,485 | 32 | 766.6 | OR915053-OR915057 | OR722514 |
| *Thesium decaryanum* | 53,949,512 | 6,909.2 | 14,264.2 | OR712484 | OR722515 |
| *Viscum trachycarpum* | 146,256,820 | 569.4 | 871.9 | OR712485 | OR722516 |
| *Ximenia americana* | 12,581,124 | 580.2 | 4,319.8 | OR712486 | OR722517 |

* complete plastomes not assembled
