# Supplementary material for: Plastome evolution in Santalales involves relaxed selection prior to loss of ndh genes and major boundary shifts of the inverted repeat
Source: Ann Bot. 2024 Aug 30;135(3):515–30. doi: 10.1093/aob/mcae145 (PMC11897430; doi:10.1093/aob/mcae145)
Supplement: mcae145_suppl_Supplementary_Table_S3 [file mcae145_suppl_supplementary_table_s3.docx]

**Table S3. Genes obtained in partially assembled plastomes.**

| **Family** | **Species** | **rRNA genes** | **tRNA genes** | **Protein coding genes** |
| --- | --- | --- | --- | --- |
| Aptandraceae | *Phanerodiscus capuronii* | *rrn16, rrn23, rrn4.5, rrn5* | *trnA-ugc, trnC-gca, trnD-guc, trnE-uuc, trnF-gaa, trnFm-cau, trnG-gcc, trnG-ucc, trnH-gug, trnI-cau, trnI-gau, trnK-uuu, trnL-caa, trnL-uaa, trnL-uag, trnM-cau, trnN-guu, trnP-ugg, trnQ-uug, trnR-acg, trnR-ucu, trnS-gcu, trnS-gga, trnS-uga, trnT-ggu, trnT-ugu, trnV-gac, trnV-uac, trnW-cca, trnY-gua* | *accD, atpA, atpB, atpE, atpF, atpH, atpI, ccsA, cemA, clpP1, infA, matK, pbf1, petA, petB, petD, petG, petL, petN, psaA, psaB, psaC, psaI, psaJ, psbA, psbB, psbC, psbD, psbE, psbF, psbH, psbI, psbJ, psbK, psbL, psbM, psbT, psbZ, rbcL, rpl14, rpl16, rpl2, rpl20, rpl22, rpl23, rpl33, rpl36, rpoA, rpoB, rpoC1, rpoC2, rps11, rps12, rps14, rps15, rps16, rps18, rps19, rps2, rps3, rps4, rps7, rps8, ycf1, ycf2, ycf3, ycf4* |
| Coulaceae | *Coula edulis* | *rrn16, rrn23, rrn4.5, rrn5* | *trnA-ugc, trnC-gca, trnD-guc, trnE-uuc, trnF-gaa, trnFm-cau, trnG-gcc, trnG-ucc, trnI-cau, trnI-gau, trnK-uuu, trnL-caa, trnL-uaa, trnL-uag, trnM-cau, trnN-guu, trnP-ugg, trnQ-uug, trnR-acg, trnR-ucu, trnS-gcu, trnS-gga, trnS-uga, trnT-ggu, trnT-ugu, trnV-gac, trnV-uac, trnW-cca, trnY-gua* | *accD, atpA, atpB, atpE, atpF, atpH, atpI, ccsA, cemA, clpP1, infA, matK, ndha, ndhb, ndhc, ndhd, ndhe, ndhf, ndhg, ndhh, ndhi, ndhj, ndhk, pbf1, petA, petB, petD, petG, petL, petN, psaA, psaB, psaC, psaI, psaJ, psbA, psbB, psbC, psbD, psbE, psbF, psbH, psbI, psbJ, psbK, psbL, psbM, psbT, psbZ, rbcL, rpl14, rpl16, rpl2, rpl20, rpl22, rpl23, rpl32, rpl33, rpl36, rpoA, rpoB, rpoC1, rpoC2, rps11, rps12, rps14, rps15, rps16, rps18, rps19, rps2, rps3, rps4, rps7, rps8, ycf1, ycf2, ycf3, ycf4* |
| Erythropalaceae | *Brachynema ramiflorum* | *rrn16, rrn23, rrn4.5, rrn5* | *trnA-ugc, trnC-gca, trnD-guc, trnE-uuc, trnF-gaa, trnFm-cau, trnG-gcc, trnG-ucc, trnH-gug, trnI-cau, trnI-gau, trnK-uuu, trnL-caa, trnL-uaa, trnL-uag, trnM-cau, trnN-guu, trnP-ugg, trnQ-uug, trnR-acg, trnS-gcu, trnS-gga, trnS-uga, trnT-ugu, trnV-gac, trnV-uac, trnW-cca, trnY-gua* | *accD, atpA, atpB, atpE, atpF, atpH, atpI, ccsA, cemA, clpP1, infA, matK, ndha, ndhb, ndhc, ndhd, ndhe, ndhf, ndhg, ndhh, ndhi, ndhj, ndhk, pbf1, petA, petB, petD, petG, petL, petN, psaA, psaB, psaC, psaI, psaJ, psbA, psbB, psbC, psbD, psbE, psbF, psbH, psbI, psbJ, psbK, psbL, psbM, psbT, psbZ, rbcL, rpl14, rpl16, rpl2, rpl20, rpl22, rpl23, rpl33, rpl36, rpoA, rpoB, rpoC1, rpoC2, rps11, rps12, rps14, rps15, rps16, rps18, rps19, rps2, rps3, rps4, rps7, rps8, ycf1, ycf2, ycf3, ycf4* |
| Erythropalaceae | *Heisteria densifrons* | *rrn16, rrn23, rrn4.5, rrn5* | *trnA-ugc, trnC-gca, trnD-guc, trnE-uuc, trnF-gaa, trnFm-cau, trnG-gcc, trnG-ucc, trnH-gug, trnI-cau, trnI-gau, trnK-uuu, trnL-caa, trnL-uaa, trnL-uag, trnM-cau, trnN-guu, trnP-ugg, trnQ-uug, trnR-acg, trnR-ucu, trnS-gcu, trnS-gga, trnS-uga, trnT-ggu, trnT-ugu, trnV-gac, trnV-uac, trnW-cca, trnY-gua* | *accD, atpA, atpB, atpE, atpF, atpH, atpI, ccsA, cemA, clpP1, infA, matK, ndha, ndhb, ndhb, ndhc, ndhd, ndhe, ndhf, ndhg, ndhh, ndhi, ndhj, ndhk, pbf1, petA, petB, petD, petG, petL, petN, psaA, psaB, psaC, psaI, psaJ, psbA, psbB, psbC, psbD, psbE, psbF, psbH, psbI, psbJ, psbK, psbL, psbM, psbT, psbZ, rbcL, rpl14, rpl16, rpl16, rpl2, rpl2, rpl20, rpl22, rpl23, rpl23, rpl32, rpl33, rpl36, rpoA, rpoB, rpoC1, rpoC2, rps11, rps12, rps12, rps12, rps14, rps15, rps16, rps18, rps19, rps2, rps3, rps4, rps7, rps7, rps8, ycf1, ycf1, ycf2, ycf2, ycf3, ycf4* |
| Erythropalaceae | *Maburea trinervis* | *rrn16, rrn23, rrn4.5, rrn5* | *trnA-ugc, trnC-gca, trnD-guc, trnE-uuc, trnF-gaa, trnFm-cau, trnG-gcc, trnG-ucc, trnH-gug, trnI-cau, trnI-gau, trnK-uuu, trnL-caa, trnL-uaa, trnL-uag, trnM-cau, trnN-guu, trnP-ugg, trnQ-uug, trnR-acg, trnR-ucu, trnS-gcu, trnS-gga, trnS-uga, trnT-ggu, trnT-ugu, trnV-gac, trnV-uac, trnW-cca, trnY-gua* | *accD, atpA, atpB, atpE, atpF, atpH, atpI, ccsA, cemA, clpP1, infA, matK, ndha, ndhb, ndhc, ndhd, ndhe, ndhf(partial), ndhg, ndhh, ndhi, ndhj, ndhk, pbf1, petA, petB, petD, petG, petL, petN, psaA, psaB, psaC, psaI, psaJ, psbA, psbB, psbC, psbD, psbE, psbF, psbH, psbI, psbJ, psbK, psbL, psbM, psbT, psbZ, rbcL, rpl16, rpl2, rpl20, rpl22, rpl23, rpl32, rpl33, rpl36, rpoA, rpoB, rpoC1, rpoC2, rps11, rps12, rps14, rps15, rps16, rps18, rps19, rps2, rps3, rps4, rps7, rps8, ycf1, ycf2, ycf3, ycf4* |
| Olacaceae | *Olax scandens* | *rrn16, rrn23, rrn4.5, rrn5* | *trnA-ugc, trnC-gca, trnD-guc, trnE-uuc, trnF-gaa, trnFm-cau, trnG-gcc, trnG-ucc, trnH-gug, trnI-cau, trnI-gau, trnK-uuu, trnL-caa, trnL-uaa, trnL-uag, trnM-cau, trnN-guu, trnP-ugg, trnQ-uug, trnR-acg, trnR-ucu, trnS-gcu, trnS-gga, trnS-uga, trnT-ggu, trnV-gac, trnV-uac, trnW-cca, trnY-gua* | *accD, atpA, atpB, atpE, atpF, atpH, atpI, ccsA, cemA, clpP1, matK, pbf1, petA, petB, petD, petG, petL, petN, psaA, psaB, psaC, psaI, psaJ, psbA, psbB, psbC, psbD, psbE, psbF, psbH, psbI, psbJ, psbK, psbL, psbM, psbT, psbZ, rbcL, rpl14, rpl16, rpl2, rpl20, rpl22, rpl23, rpl32, rpl33, rpl36, rpoA, rpoB, rpoC1, rpoC2, rps11, rps12, rps14, rps15, rps16, rps18, rps19, rps2, rps3, rps4, rps7, rps8, ycf1, ycf2, ycf3, ycf4* |
| Strombosiaceae | *Tetrastylidium peruvianum* | *rrn16, rrn23, rrn4.5, rrn5* | *trnA-ugc, trnC-gca, trnD-guc, trnE-uuc, trnF-gaa, trnFm-cau, trnG-gcc, trnG-ucc, trnH-gug, trnI-cau, trnI-gau, trnK-uuu, trnL-caa, trnL-uaa, trnL-uag, trnM-cau, trnN-guu, trnP-ugg, trnQ-uug, trnR-acg, trnR-ucu, trnS-gcu, trnS-gga, trnS-uga, trnT-ggu, trnT-ugu, trnV-gac, trnV-uac, trnW-cca, trnY-gua* | *accD, atpA, atpB, atpE, atpF, atpH, atpI, ccsA, cemA, clpP1, infA, matK, ndha, ndhb, ndhc, ndhd, ndhe, ndhf, ndhg, ndhh, ndhi, ndhj, ndhk, pbf1, petA, petB, petD, petG, petL, petN, psaA, psaB, psaC, psaI, psaJ, psbA, psbB, psbC, psbD, psbE, psbF, psbH, psbI, psbJ, psbK, psbL, psbM, psbT, psbZ, rbcL, rpl14, rpl16, rpl2, rpl20, rpl22, rpl23, rpl32, rpl33, rpl36, rpoA, rpoB, rpoC1, rpoC2, rps11, rps12, rps14, rps15, rps16, rps18, rps19, rps2, rps3, rps4, rps7, rps8, ycf1, ycf2, ycf3, ycf4* |
| Ximeniaceae | *Curupira tefeensis* | *rrn16, rrn23, rrn4.5, rrn5* | *trnA-ugc, trnC-gca, trnD-guc, trnE-uuc, trnF-gaa, trnFm-cau, trnG-gcc, trnG-ucc, trnH-gug, trnI-cau, trnI-gau, trnK-uuu, trnL-caa, trnL-uaa, trnL-uag, trnM-cau, trnN-guu, trnP-ugg, trnQ-uug, trnR-acg, trnR-ucu, trnS-gcu, trnS-gga, trnS-uga, TrnT-ggu, TrnT-ugu, trnV-gac, trnV-uac, trnW-cca, trnY-gua* | *accD, atpA, atpB, atpE, atpF, atpH, atpI, ccsA, cemA, clpP1, infA, matK, pbf1, petA, petB, petD, petG, petL, petN, psaA, psaB, psaC, psaI, psaJ, psbA, psbB, psbC, psbD, psbE, psbF, psbH, psbI, psbJ, psbK, psbL, psbM, psbT, psbZ, rbcL, rpl14, rpl16, rpl2, rpl20, rpl22, rpl23, rpl32, rpl33, rpl36, rpoA, rpoB, rpoC1, rpoC2, rps11, rps12, rps14, rps15, rps16, rps18, rps19, rps2, rps3, rps4, rps7, rps8, ycf1, ycf2, ycf3, ycf4* |
