# Supplementary material for: Plastome evolution in Santalales involves relaxed selection prior to loss of ndh genes and major boundary shifts of the inverted repeat
Source: Ann Bot. 2024 Aug 30;135(3):515–30. doi: 10.1093/aob/mcae145 (PMC11897430; doi:10.1093/aob/mcae145)
Supplement: mcae145_suppl_Supplementary_Table_S5 [file mcae145_suppl_supplementary_table_s5.docx]

| **Species** | ***ndhA*** | ***ndhB*** | ***ndhC*** | ***ndhD*** | ***ndhE*** | ***ndhF*** | ***ndhG*** | ***ndhH*** | ***ndhI*** | ***ndhJ*** | ***ndhK*** |
| --- | --- | --- | --- | --- | --- | --- | --- | --- | --- | --- | --- |
| *Brachynema ramiflorum* | 0.6 | 0.6 | 0,1 | 0.7 | 0.3 | 0.6 | 0.8 | 0.3** | 0.3** | 0.5 | 5.2 |
| *Erythropalum scandens* | 0.8 | 1.7 | 2.1 | 0.3** | 0.7 | 0.4* | 0.3* | 0.2* | 0.6* | 0.5 | 0.0* |
| *Heisteria densifrons* | 2.3 | 1.0 | 0.0 | 0.3 | 6.3 | 0.4 | 1.1 | 0.0 | 0.7 | 0.5 | 1.6 |
| *Maburea trinervis* | 0.0** | 1.2 | 0.2 | 0.2 | 0.5 | - | 0.8 | 0.9 | 0.2** | 8.7* | 1.0 |
| *Diogoa zenkeri* | 1.2 | 1.2 | 6.1 | 1.2 | 1.2 | 0.2* | 1.2 | 47.9 | 1.1 | 1.2 | 0.0 |
| *Engomegona gordonii* | 1.3 | 1.2 | 0.0 | 0.6 | 8.3 | 0.3* | 28.6 | 0.1 | 1.1 | 0.7 | 0.6 |
| *Scorodocarpus borneensis* | 0.4 | 0.6 | 1.2 | 0.8 | 50.0 | 0.3 | 0.5 | 0.4 | 0.3 | 1.2 | 50.0 |
| *Strombosia pustulata* | 2.8 | 16.5 | 0.3 | 1.3 | 1.3 | 0.2* | 20.5 | 46.3 | 0.2* | 1.2 | 34.1 |
| *Strombosiopsis tetrandra* | 0.0** | 1.0 | 1.2 | 0.1 | 50.0 | 0.2* | 1.2 | 48.5 | 0.3 | 1.0 | 1.2 |
| *Tetrastylidium peruvianum* | 0.1 | 1.2 | 0.0 | 0.0 | 50.0 | 0.2* | 0.8 | 43.7 | 1.2 | 1.2 | 0.6 |
| *Coula edulis* | 1.4 | 1.2 | 1.1 | 0.4 | 50.0 | 1.1 | 1.2 | 42.5 | 1.2 | 0.7 | 50.0 |
| *Minquartia guianensis* | 0.3 | 0.0 | 1.0 | 0.4 | 1.2 | 1.0 | 19.3 | 45.1 | 1.2 | 1.2 | 0.6 |
| *Octoknema affinis* | 1.3 | 18.0 | 0.3 | 0.2 | 0.0 | 1.4 | 22.7 | 1.0 | 1.5 | 0.0 | 1.5 |

**Table S5.** Changes in selection pressure in 11 individual *ndh* genes. Reference group: *Crataegus marshallii*, *Helianthus annuus*, *Myoporum bontioides*, and *Silene kiusiana*. Blue boxes indicate significant relaxed selection, red boxes indicate significant intensified selection. Values are the selection intensity parameter *k*. Significance levels for *k*: * *p*<0.05, ** *p*<0.01.
